# Disentangling the role of adaptive immunity in Alzheimer’s disease

**DOI:** 10.1186/s13024-026-00985-7

**Published:** 2026-08-13

**Authors:** Marco Zattoni, Ludwig Aigner

**Affiliations:** 1 Institute of Molecular Regenerative Medicine, Paracelsus Medical University, Salzburg, Austria; 2 Austrian Cluster for Tissue Regeneration, Vienna, Austria

## Abstract

Alzheimer’s disease (AD), the most common age-related neurodegenerative disorder, is characterized by progressive synaptic and neuronal loss, amyloid-β deposition, hyperphosphorylated tau accumulation, and chronic neuroinflammation, traditionally attributed primarily to the innate immune system. Increasing evidence, however, implicates the adaptive immune system as an active contributor to AD pathogenesis and progression. This emerging perspective has stimulated the development of immunomodulatory strategies aimed at mitigating cognitive decline and neuropathological hallmarks of the disease. In this review, we examine how adaptive immune responses change throughout the course of AD, integrating findings from both human studies and animal models while emphasizing the temporal dynamics of these alterations. We critically evaluate areas of consensus and controversy in the field, highlighting how differences in experimental models, disease stages, and methodological approaches contribute to divergent findings. Finally, we assess current therapeutic strategies targeting adaptive immunity, discussing their potential mechanisms of action and identifying key therapeutic windows and routes of administration that may maximize efficacy. By providing a comprehensive overview of adaptive immune involvement in AD, this review aims to inform the development of more precise and effective immunomodulatory interventions.

## Background

Alzheimer’s disease (AD) is a neurodegenerative condition which accounts for almost two-third of all cases of dementia [1]. It is indeed the most common neurodegenerative disease in the elderly, affecting around 10% of people over 65 and around 30% of those over 85 [2]. The strongest risk factor for AD is aging [3]. Due to the worldwide increase in the elderly population, the prevalence of individuals with dementia will triple from 50 million in 2019 to 152 million by 2050 [4]. Therefore, there is an urgent need for efficient AD therapies to reduce the burden for patients and families, for society and health care system, and costs of an imminent surge in dementia cases.

AD was long defined primarily by its amyloid-β (Aβ) plaques and tau pathology, while it is by now recognized that neuroinflammation contributes to disease onset and progression [5]. This is exemplified by astrocytes and microglial activation in response to Aβ aggregates and degenerating synapses [6]. Genome-wide association studies have strengthened this view by identifying AD risk variants in genes that regulate microglial function and innate immune pathways, such as TREM2 and CD33 [7]. More recent data indicate that this innate immunitycentric perspective of neuroinflammation is by far incomplete, as it does not account for emerging findings that peripheral lymphocytes, the microbiota-gut-brain axis, external factors (e.g., injury or viral infection) and crosstalk between the central nervous system (CNS) and peripheral immunity all shape the overall inflammatory milieu in AD (see for review [8, 9]). The latter was largely neglected for many years, as the CNS was considered as an immuneprivileged site that was largely shielded from adaptive immune surveillance [10, 11]. This concept has been fundamentally revised with the discovery of functional meningeal lymphatic vessels [12, 13], with extensive evidence for continuous trafficking of immune cells across CNS borders [14–16], with evidence for the adaptive immune system being required for different brain functions [17–20], and with lessons learned from neuroimmunology and autoimmune diseases such as multiple sclerosis (MS) (see for review [21, 22]). Collectively, these advances, together with the growing knowledge of AD as a systemic immunological disorder involving dynamic communication between CNS-resident innate immune cells and adaptive immune populations in the periphery, operating in the CNS border immune niches across the blood–brain barrier (BBB), meninges and meningeal lymphatic system (see for review [23, 24]), are helping to improve our understanding of the disease. Further experiments investigating the interaction between specific T cell and B cell subsets, and microglia, astrocytes and aggregated Aβ/tau are now central to deciphering novel disease mechanisms and variability in clinical trajectories. This evolving view provides the conceptual basis for immunomodulatory and vaccination-based strategies in AD, but it also underscores the need to distinguish beneficial from detrimental adaptive responses to avoid off-target neurotoxicity in future therapies.

Modulation of adaptive immune cells in AD animal models has yielded variable outcomes depending on the model used, disease stage, therapeutic strategy, and targeted cell type. Precise tuning of adaptive immune responses in accordance with disease progression and the selection of appropriate animal models is crucial for the development of innovative AD therapies. Investigating the timing and delivery methods of treatments targeting adaptive immunity holds promise for devising more personalized approaches to managing disease progression in AD. In view of the paucity of extensive reviews that have critically addressed the modulation of adaptive immunity in animal models of AD, this comprehensive review aims to fill that gap.

## The entry routes of adaptive immune cells into the AD brain

The main route used by peripheral lymphocytes to infiltrate brain parenchyma is the BBB, which is damaged in cases of AD pathology [25, 26]. This might occur as a consequence of microglia-mediated release of pro-inflammatory cytokines, which promote for examples endothelial cells to produce chemokine (C-X-C motif) ligand (CXCL) 8, a chemokine that binds to the chemokine CXC receptor (CXCR) 2 enriched on T cells [27]. An alternative mechanism occurs via the vascular cell adhesion molecule 1 (VCAM-1)–α4β1 integrin interaction between brain endothelial vessel and leukocytes [28]. Also, the transmigration of lymphocytes across the BBB might be involved in Aβ pathology. Indeed, Aβ-mediated upregulation of the chemokine receptor CCR5, which is known to be expressed in brain endothelial cells during the late stage of AD, has been demonstrated to promote T cell transmigration in vitro. Although this pathway has not been proven to occur in humans, the results suggest a potential for Aβ- and CCR5-dependent T cell recruitment into the brain [29]. Similarly, Aβ_42_ can activate the microglial release of Tumor necrosis factor α (TNF-α), which in turn promotes the trans endothelial migration of T cells to the parenchyma [30].

In addition to the BBB, lymphocytes use other entry sites during AD progression. These include the choroid plexus, which is located within the blood-cerebrospinal fluid barrier [31, 32] and the dura mater/blood-meningeal barrier (see for review [33, 34]). A recent analysis of meningeal dural sinuses has revealed the presence of ectopic lymphoid structures (ELS) in various animal models of AD. These structures bear a resemblance to secondary lymphoid organs and contain both T and B cells. The data indicates that the presence of ELS is influenced by factors such as sex, age, and, most notably, the type of AD pathology (i.e., a higher prevalence observed in amyloidosis compared to tauopathy) [35]. This suggests the necessity for a meticulous experimental design to ascertain the role of this structure and, more broadly, of adaptive immunity in the progression of AD.

## Lessons learned from targeting the broad spectrum of adaptive immunity

Groundbreaking work carried out on transgenic mice revealed that adaptive immune cells play an important role in modulating AD pathology (Table 1). Breeding the 5×FAD mouse model (carrying Swedish (K670N/M671L), Florida (I716V), and London (V717I) mutations in human amyloid precursor protein, APP, and the M146L and L286V mutations in presenilin gene PSEN1, all associated with familial cases of AD) with Rag2^-/-^/Il2rg^-/-^ mice, which lack functional T and B cells, resulted in an increase in Aβ plaques load, higher cytokine production and elevated numbers of activated CD68^+^ microglia cells [36, 37]. This phenotype has been rescued by pre-immune immunoglobulin (Ig) G treatment, indicating the protective role of adaptive immunity in AD pathogenesis [36]. Notably, the intravenous (i.v.) injection of IgG antibody in 3×TgAD animals (carrying APP Swedish mutation, the M146V in PSEN1, and the P301L in tau gene) improved memory in the novel object recognition (NOR) test. This effect was accompanied by a reduction in the soluble Aβ_42_/Aβ_40_ ratio and a decrease of Aβ oligomer levels. There was only a limited effect on phosphorylated tau (pTau), which suggests that IgG may play a protective role primarily in AD-related amyloidosis [42].

**Table 1:** Studies focusing on targeting the broad spectrum of adaptive immunity

Animal model	Intervention	Age of the intervention	Effects on the adaptive immune system	End of the experiment	Effects on AD-related pathology	References
5×FAD	Crossbreeding with Rag2^-/-^/IL2ry^-/-^mice	NA	Lacked both B and T cells	7 months	Detrimental: ↑ soluble and insoluble Aβ and Aβ plaques load ↓ crosstalk between adaptive and innate immunity from RNA-seq analysis ↑ microglia number (but ↓ process length and branching) and ↓ microglia phagocytic activity ↑ IL-1β, IL-6 and TNF-α NOTE: IgG treatment and BM adoptive transfer rescued Aβ pathology	[36]
5×FAD	Crossbreeding with Rag2^-/-^/IL2ry^-/-^mice	NA	Lacked both B and T cells	10 months	Detrimental: ↑ Aβ plaques area Comparable number of GFAP^+^ and Iba1^+^ cells, but ↑ number of CD68^+^ microglia cells	[37]
APPswe/PSEN1dE9	Crossbreeding with Rag2^-/-^ mice	NA	Altered B and T cells maturation	3–8 months	Beneficial: Unaltered spatial working memory (in the Y-maze test) and recognition memory (in the NOR test) ↓ soluble and insoluble Aβ and amyloid plaques (only at 8 months) ↓ GFAP^+^ cell number (at 8 months) Unaltered microgliosis	[38]
APP^N-L-GF^	Crossbreeding with Rag2^-/-^ mice	NA	Lacked mature adaptive immune cells (CD45^+^CD3^+^ T cells and CD45^+^CD19^+^ B cells)	5–6 months	Beneficial: ↑ spatial learning in the MWM test and ↓ freezing in the anxiety-related behavior test (no changes in the spatial working memory in the elevated open T-maze test) ↓ Aβ plaque load and insoluble Aβ_42_ ↓ Iba1^+^ cells, but with ↑ branch number ↓ GFAP^+^ cells ↑ NeuN^+^ cells and PSD95^+^ signal	[39]
APP^N-L-GF^	Crossbreeding with Rag1^-/-^ mice	NA	Lacked functional lymphocytes ↓ brain-resident CD8^+^ T cells	12 months	Beneficial: ↓ myelin abnormalities, ↓ number of BCAS1^+^ swellings and EdU^+^Olig2^+^ cells ↓ MHC-II^+^Iba1^+^ cells	[40]
APPswe/PSEN1dE9	Fingolimod administration via drinking water (for two months)	8 months	↓ brain B and T lymphocytes ↓ biological processes related to B cell activation and differentiation and T cell-mediated immunity	10 months	Beneficial: ↑ cognitive deficits in the MWM and the Y-maze tests ↓ molecular pathways associated with neurodegenerative diseases, immune system and aging ↓ Aβ deposition, and Aβ_40_ and Aβ_42_ levels ↑ CTF-α, ↓ CTF-β, ↓ BACE1 (no alteration in ADAM10 and PSEN1) ↓ total tau and pTau at Thr181, Ser199, Thr231, Ser396 and Ser404 ↓ CD68^+^ and GFAP^+^ cells ↓ TNF-α (unaltered levels of IL-1β) ↑ Map2^+^ staining in the hippocampal CA1 region ↓ activated Caspase-3 in the CA3 region ↑ synapse-related proteins (PSD95, SNAP25 and VAMP1)	[41]

The table includes animal models, interventions and the age at which these are implemented (where applicable otherwise stated as not applicable, NA), the effects on the adaptive immune system, the age at which behavioral tests and sacrifice are performed, and the effects on AD-related pathology

However, studies that exploited the crossbreeding of different AD transgenic mouse models with animals characterized by a defective adaptive immune system came to the opposite conclusion. Although no changes in the number of Iba1^+^ microglia cells or cognitive improvements were observed in APPswe/PSEN1dE9 (carrying APP Swedish mutation and a deletion of exon 9 in PSEN1 gene) mice crossbred with Rag2^-/-^ animals, they were characterized by reduction in Aβ pathology and astrogliosis. Furthermore, the adoptive transfer of bone marrow (BM) cells from both wild type (WT) and Rag2^-/-^ animals into irradiated APPswe/PSEN1dE9 animals confirmed the detrimental effect of a functional adaptive immune system during AD progression. Brain neuropathology of the animals adoptively transferred with Rag2^-/-^ cells revealed a reduction of Aβ plaques load, decreased insoluble Aβ_40_ and Aβ_42_, and increased Aβ phagocytosed materials by microglia cells, suggesting an enhancement of Aβ clearance in the absence of functional T and B cells [38].

Similarly, the absence of adaptive immune responses lowered cognitive decline and memory loss in APP^NL-G-F^ (carrying a humanized Aβ region, includes the Swedish, the I716F, and the E693G mutations) cross with Rag2^*−/−*^ mice. Cortex and hippocampal region of these transgenic animals showed a reduction of Aβ pathology, microgliosis and astrocytosis [39]. A similar detrimental effect of the adaptive immune system was observed by breeding APP^NL-G-F^ with Rag1^-/-^ animals, which are characterized by an early arrest of T and B cell development as Rag2^-/-^ mice. Although the cortical region of double transgenic animals showed no alteration in Aβ plaques load, a reduction in Aβ pathology-related myelin abnormalities has been observed [40]. These discrepancies could be attributed to the different AD transgenic animal models used for crossbreeding experiments. The 5×FAD animals used in Marsh et al., 2016 [36] and Faridar et al., 2022 [37] are characterized by an accelerated AD pathology compared to the APPswe/PSEN1dE9 used in Späni et al., 2015 [38], pointing toward a temporal relation between the response of adaptive immune system and the appearance of Aβ pathology. Furthermore, while the Rag2^-/-^/Il2rg^-/-^ model used by Marsh et al., 2016 [36] also lacks natural killer (NK) cells, the Rag2^-/-^ mice used by Späni et al., 2015 [38] suggest that the absence of NK cells may be potential confounding factor to be taken into account when interpreting these data. Adaptive immunity might play a protective role during a robust and abrupt AD pathology, but it may show harmful effect in the course of slower disease progression. This hypothesis is partially supported by the results from Van Hoecke et al., 2024 [39] and Kedia et al., 2024 [40]. Indeed, the emergence of brain amyloidosis of the APP^NL-G-F^ mice is slightly delayed compared to 5×FAD animals [43], suggesting that the absence of a functional adaptive immune system in APP^NL-G-F^ mice has no influence on Aβ pathology progression, because of a temporal disparity in Aβ plaques accumulation. These results suggest that differences in the adaptive immune system between transgenic animal models must be considered alongside the temporal aspect when studying AD pathology. Furthermore, all the aforementioned studies primarily discuss findings from developmental immune deficiencies that persist throughout the mouse’s life (achieved by crossbreeding with Rag1 and Rag2 knockout animals). This limits the interpretability of the role of the adaptive immune system during aging, which is considered one of the major risk factors for the progression of AD.

As an alternative to genetic models, fingolimod has been used to reduce the infiltration of the brain by T and B lymphocytes. Fingolimod is a drug that acts as a functional antagonist of the sphingosine-1-phosphate (S1P) receptor, impeding the exit of lymphocytic cells from the lymph node [44]. The APPswe/PSEN1dE9 treated mice showed better performance in the Morris Water Maze (MWM) and Y-maze tests, had less amyloid plaque, less pTau and neuroinflammation, and had healthier neurons [41]. Although these results supported a harmful role for both T and B lymphocytes, the S1P receptors targeted by fingolimod are also expressed on CNS-resident cells and innate immune populations [45]. Therefore, the reported benefits may not reflect purely adaptive immune modulation. Consequently, an analysis of the adaptive immune system at the level of individual cell populations is required to examine the various aspects of adaptive immunity in AD.

## The role of B cells in AD pathogenesis

B lymphocytes develop from hematopoietic stem cells in the BM and once the rearrangement process of the Ig gene segments occurs, immature B cells leave the BM and migrate to peripheral lymphoid organs, where they might differentiate into plasma cells (PC) secreting antibodies [46]. It is hypothesized that B cells found in the CNS originate from the adjacent skull BM and enter the dura through transosseous channels. In this instance, the formation of ELS occurs, thereby supporting local germinal center reactions that generate IgG^+^ PCs. Conversely, IgA^+^ PCs accumulate in the peri-sinus region via the bloodstream [47]. 

Although a growing body of literature has recently started to focus on the role of B cells in AD, studies on brain-infiltrating B lymphocytes are limited and contradictory. Despite a study stating the presence of IgG^+^ neurons containing intracellular Aβ_42_ deposits in AD brains [48], no studies, to our knowledge, revealed the presence of B cells in the parenchyma of human AD cases. Nevertheless, B cells have been detected in the brain of transgenic animal models. CD19^+^ and B220^+^ B cells appeared in frontal cortex and meninges of 3-month-old 5×FAD mice, but the number of CD19^+^ B cells decreased at later stages when compared to WT animals [49, 50]. Spatial transcriptomic analysis revealed the enrichment of B cell signatures in the brain of the 7-month-old 5×FAD mice [51]. Furthermore, 9-month-old 5×FAD mice showed a significantly higher number of CD19^+^ B lymphocytes in the meninges compared with age-matched WT mice [52]. Flow cytometric analysis of BM immune cells and tracing of skull-derived cells indicated that BM-derived B lymphocytes infiltrate the brain parenchyma of both 3-month-old 5×FAD and 8-month-old APPswe/PSEN1dE9 mice [53]. This suggests that B lymphocytes infiltrate the brain parenchyma in a time-dependent manner in different AD animal models. More recently, a potential lung-brain axis has been proposed as alternative mechanism responsible for B cell parenchymal recruitment. Indeed, CD19^+^ B cells derived from the lungs of 5×FAD mice exhibited enhanced migration towards the CNS one day after retro-orbital transplantation in comparison to those derived from WT mice. Subtype population analysis following intrathecal injection of fluorescently labelled cells has revealed that, although both memory B (Bmem) cells and PCs are significantly increased in the lungs, meninges and frontal cortex of 3-month-old 5×FAD mice, a greater number of Bmem (CD19^+^CD38^-^CD27^+^) cells accumulate in the meninges and cortex compared to PCs (CD19^+^CD138^+^). Single-cell RNA sequencing (scRNA-seq) and immunofluorescence analysis have suggested that the axis involved in the recruitment process occurs via CXCL12-expressing microglia and CXCR4-expressing Bmem cells [54].

However, the literature contains contradictory results regarding the role of brain-resident B cells in AD pathology (Table 2). 5×FAD mice that were deficient in mature B cells (muMT^-/-^) exhibited deficits in short-term memory in the NOR and Y-maze tests, as well as dysfunctions in long-term memory in the Barnes maze test. Under a neuropathological point of view, 5×FAD/muMT^-/-^ mice showed increased Aβ plaques accumulation, together with increased number of GFAP^+^ and CD86^+^Iba1^+^ cells and an augmented neurodegenerative-associated pathology, as resulted by the loss of synaptophysin (SYP) and postsynaptic density protein 95 (PSD95) signals in brain tissue [50]. These data are corroborated by antibody-mediated depletion of mature B cells, via intraperitoneal (i.p.) injection of anti-CD20 antibody, or antibody-mediated neutralization of interleukin (IL)-35, mainly produced by B lymphocytes [58]. Mechanistically, when recombinant IL-35 is intracerebroventricular (i.c.v.) injected in 5×FAD/muMT^-/-^ mice, the memory deficits, the loss of SYP and PSD95, and the Aβ pathology are rescued, suggesting a B cells-independent beneficial effect of IL-35 [50]. A similar protective role of B lymphocytes during AD pathology is supported by the antibody-mediated depletion of B cells (via i.p. injection of anti-CD19 and anti-B220) performed in APP/PS1 animals. The results showed that the animals treated performed worse in the MWM and open field (OF) tests and displayed poorer working memory in the Y-maze test. Furthermore, B cell-depletion increased Aβ deposition in brain tissue [55]. In a similar manner, the reduction of peripheral B cells in 4-month-old APPswe/PSEN1dE9 animals by splenectomy led to impaired spatial learning ability in MWM test and worsened short-term memory deficits in the NOR test. Treated mice showed increased number of Aβ plaques, activated Iba1^+^CD68^+^ microglia and astrocytes, augmented pTau and a decrease in brain MAP2^+^ and NeuN^+^ neuronal cells [56].

**Table 2:** Studies focusing on targeting B cells

Animal model	Intervention	Age of the intervention	Effects on the adaptive immune system	End of the experiment	Effects on AD-related pathology	References
5×FAD	crossbreeding with muMT^-/-^	NA	Lacked mature B cells	3 months	Detrimental: ↓ short-term memory in the NOR and the Y-maze tests, and ↓ long-term memory in the Barnes maze test ↑ Aβ plaques accumulation ↑ GFAP^+^ and CD68^+^Iba1^+^ cells ↓ levels of SYP and PSD95 proteins ↑ expression of *Bace1* NOTE: rescued deficits upon IL-35 treatment	[50]
5×FAD	i.p. injection of anti-CD20 (once a week for one month)	2 months	↓ B cells	3 months	Detrimental: ↓ short-term memory in the NOR and the Y-maze tests and ↓ long-term memory in the Barnes maze test ↑ Aβ plaques accumulation ↑ number of GFAP^+^ and CD68^+^Iba1^+^ cells ↓ levels of SYP and PSD95 proteins	[50]
APP/PS1 (mutations not specified)	i.p. injection of anti-CD19 and anti-B220 antibodies (every five days for three months)	4 months	↓ B cells	7 months	Detrimental: ↓ cognitive function in MWM and OF tests, and ↓ working memory in the Y-maze test ↑ Aβ plaques burden in the cortex and the hippocampus	[55]
APPswe/PSEN1dE9	Splenectomy	4 months	↓ peripheral B cells (no changes in T cells)	9 months	Detrimental: ↓ spatial learning ability in the MWM test and ↓ short-term memory recognition in the NOR test ↑ Aβ plaques number and more compact plaques around vessel, and levels of soluble and insoluble Aβ _40_ and Aβ_42_ ↑ pTau (T231) ↑ TNF-α and IL-6 ↓ MAP2^+^ and NeuN^+^ cells and ↑ level of apoptosis ↑ GFAP^+^ cells and activated Iba1^+^CD68^+^ microglia	[56]
3×Tg-AD	crossbreeding with J_H_T mice	NA	Lacked B cells	12–15 months	Beneficial: ↑ learning memory in the MWM test ↓ Aβ plaques and ↓ soluble Aβ_42_ and Aβ_40_ levels ↑ number of large ameboid microglia	[49]
APPswe/PSEN1dE9	crossbreeding with J_H_T mice	NA	Lacked B cells	5–9 months	Beneficial: ↑ learning memory in the MWM test ↓ Aβ plaques ↑ TGFβ^+^ microglia (but no changes in the overall microglia number)	[49]
5×FAD	i.p. injection of antiCD20/B220	9–11 months	↓ B cells	11–13 months	Beneficial: ↑ distance travel in OF test ↓ Aβ plaques ↓ large size microglial cells (but unaltered TGFβ^+^ and IL-10^+^ microglial cells)	[49]
3×Tg-AD	i.p. injection of antiCD20/B220	15–18 months	↓ B cells	17–20 months	Beneficial: ↓ Aβ plaques ↑ TGFβ^+^ and IFN-γ^+^ microglia cells (but unaltered ameboid microglial cells)	[49]
APPswe/PSEN1dE9	i.c.v. injection of BM-derived ABCs (CD19^+^CD11c^+^) from 18-month-old 5×FAD once	10 months	↑ brain-resident B cells	11 months	Detrimental: ↑ Aβ plaque content in the hippocampus and the cortex ↑ soluble Aβ_42_ ↑ reactive microgliosis and ↓ complexity of the microglial branches	[53]
5×FAD	i.c.v. injection of BM-derived ABCs (CD19^+^CD11c^+^) from 18-month-old 5×FAD once a month	2.5 months	↑ brain-resident B cells	5 months	Detrimental: ↓ spatial learning and memory performance the MWM test, and ↓ performance in the Y-maze test ↑ Aβ plaque content in the hippocampus and cortex and ↑ level of soluble Aβ_42_ ↑ reactive microgliosis and ↓ complexity of the microglial branches ↓ homeostatic cluster and ↑ disease-associated microglia cluster, inflammation and IFN-responsive cells cluster, together with ↑ expression of disease-related marker genes (from scRNA-seq analysis on sorted CD11b^+^ cells) ↓ P2RY12 (homeostatic marker) and ↑ CD11c (disease-related marker)	[53]
TAU58/2^tg/wt^	crossbreding with muMT^-/-^ mice	NA	Lacked mature B cells in the brain and the spleen	4–8 months	Detrimental: ↓ learning memory in the MWM test, but unaltered anxiety/disinhibition in the EPM test and in motor abilities in the rotarod test No changes in GFAP^+^ and Iba1^+^ cells ↓ pS422^+^ tau cells in amygdala Slight changes in tau solubility	[57]
TAU58/2^tg/wt^	i.v. injection ofanti-CD20 (fortnightly for six months and a half)	3 weeks	↓ splenic IgM^+^/B220^high^ B cells	3.5–7 months	No major effects: Unaltered spatial learning in the MWM test, in anxiety/disinhibition in the EPM test and in motor abilities in the rotarod test No changes in GFAP^+^ and Iba1^+^ cells ↑ pS396/pS404 tau in the hippocampus Slight changes in tau solubility	[57]
5×FAD	intratracheal instillation of AAV5 encoding mouse B-cell activating factor	7 months	↑ B cells in the frontal cortex	10 months	Beneficial: ↑ short-term spatial working memory in the Y-maze test and ↓ spatial learning and memory in the Barnes maze test (no change in the NOR test) ↓ BACE1 ↓ Thioflavin S (Thio-S^+^ and 6E10^+^ Aβ plaques ↓ Iba1^+^ and GFAP^+^ area ↑ SYP, synapsin (SYN) and PSD-95 in the frontal cortex	[54]
5×FAD	intratracheal instillation of anti-CD40L	3 months	↓ B cells in the frontal cortex and meninges	14 d.p.i	Detrimental: ↓ learning and declarative/episodic-like memory in the NOR test, ↓ short-term spatial working memory in the Y-maze test and ↑ spatial learning and memory in the Barnes maze test ↑ Thio-S^+^ and 6E10^+^ Aβ plaques ↑ Iba1^+^ and GFAP^+^ area	[54]
5×FAD	intratracheal instillation of CD27^+^ Bmem cells isolated from the lungs of 3-month-old 5×FAD mice	7 months	↑ CD27^+^ Bmem cells in the frontal cortex and the lungs ↑ expression levels of IL-35 secreted by Bmem cells	14 d.p.i	Beneficial: ↑ learning and declarative/episodic-like memory in the NOR test, ↑ short-term spatial working memory in the Y-maze test and ↓ spatial learning and memory in the Barnes maze test ↓ Thio-S^+^ and 6E10^+^ Aβ plaques ↓ Iba1^+^ and GFAP^+^ area	[54]
5×FAD	i.p. injection ofanti-CD20 (once a week and lasted for one month)	2 months	↓ Bmem cells (not PCs) in the meninges, the frontal cortex, and the lungs	3 months	Detrimental: ↑ Thio-S^+^ and 6E10^+^ Aβ plaques ↑ Iba1^+^ area	[54]

The table includes animal models, interventions and the age at which these are implemented (where applicable, otherwise stated as not applicable, NA), the effects on the adaptive immune system, the age at which behavioral tests and sacrifice are performed, and the effects on AD-related pathology. d.p.i: days post injection

In contrast, when APPswe/PSEN1dE9 are crossed with B cell-deficient mice (J_H_T), the resulting double transgenic animals showed improved learning deficiency in the MWM test. The decrease in soluble Aβ_40_ and Aβ_42_, as well as the reduction in Aβ plaque load observed in 3×Tg-AD/J_H_T mice, supports the theory that B lymphocytes contribute to the development of AD. Consistent with these findings, depleting B cells in 9–11-month-old 5×FAD mice using anti-CD20 and anti-B220 antibodies improved performance in the OF test. Analysis of the brain tissue of B-cell-depleted animals revealed fewer Aβ plaques and microglia. Similarly, analysis of the brain tissue of 15- to 18-month-old 3×Tg-AD mice that had been treated with an anti-CD20/B220 antibody showed a reduction in Aβ plaque load [49].

A detailed analysis of B cells infiltrating the brains of 5×FAD animals was conducted, revealing a peculiar age-associated signature (CD45^+^CD19^+^CD11b^+^CD11c^+^CD21^−^CD23^−^, referred to as ABCs). When BM-derived ABCs (CD19^+^CD11c^+^) from 10-month-old 5×FAD mice were injected into the lateral ventricles, the animals showed a worsening cognitive function compared to 5×FAD mice that received control cells. Neuropathologically, this was accompanied by increased Aβ pathology and microgliosis, as evidenced by scRNA-seq data on sorted CD11b^+^ cells. The same pathological changes were replicated in 10-month-old APPswe/PSEN1dE9 animals that received ABCs from 18-month-old animals [53].

Recently, intratracheal adeno associated virus (AAV) delivery of B-cell activating factor (BAFF), a cytokine that is critical for the survival, maturation, and homeostasis of B cells [59], in 7-month-old mice resulted in enhanced cognitive function in the Y-maze and Barnes maze tests. In addition, this treatment led to a reduction in Aβ plaque deposition and an increase in synapse-related protein expression. To demonstrate the protective function of B lymphocytes, with a particular emphasis on those of lung origin, in the progression of AD, the authors instilled an anti-CD40L antibody, known to specifically destroy GCs in the lung lymph nodes [60], into the trachea of 3.5-month-old 5×FAD mice. This resulted in the occurrence of short- and long-term working memory deficits, as well as increased Aβ deposition and glial activation in the frontal cortex. Nevertheless, when both treatments were performed intrabronchially, no effect on Aβ deposition was observed, suggesting that the effect was anatomical-dependent. To investigate the specific subtypes of B cells involved in AD-like pathology, the authors intratracheally administered CD27^+^ Bmem cells isolated from the lungs of 3-month-old 5×FAD mice. This treatment resulted in improvements in both short- and long-term working memory in the 5×FAD mice, as well as a reduction in Aβ deposition and microglia activation in the frontal cortex. The present findings are corroborated by the i.p. delivery of CD20-neutralising antibodies into 2-month-old 5×FAD mice, which led to a specific reduction in the number of Bmem cells in the lungs and brain tissue and reduced Aβ deposition and microglial activation in the frontal cortex [54]. These results suggest that lung Bmem cells may play a role in counteracting the pathological progression associated with AD. In relation to pure tau pathology, it appears that B lymphocytes fulfil a time-dependent role in the associated functional deficits. Indeed, while TAU58/2 mice (expressing one copy of the four-repeat tau gene with the P301S mutation, associated with genetic form of frontotemporal dementia) crossed with muMT^-/-^ showed a moderate delayed learning in the MWM task, brain neuropathology revealed a slight effect on tau protein phosphorylation (at serine 422) and solubility, with no alterations to the glial response. Similarly, altered tau solubility was evident in antibody-mediated B cell depletion in the same animal model; however, no effect on cognitive function was observed, suggesting that only the absence of B cells throughout life affects tau-related cognitive deficits [57]. However, further experimentation is required to determine the role of B cells in tauopathy and thereby resolve this potential time-dependent discrepancy. The contrasting results observed in the crossbreeding experiments conducted by Kim et al., 2021 [49] and Feng et al., 2023 [50] could be attributed to the different AD animal models employed (APPswe/PSEN1dE9 versus 5×FAD, respectively). Similarly, antibody-mediated B cells depletion led to different conclusions, since while in Kim et al*.,* 2021 [49] 3×TgAD and 5×FAD animals were used, Xiong et al*.,* 2021 [55] took advantage of the APP/PS1 model. However, the contrasting results obtained by Feng et al., 2023 [50] and Kim et al*.,* 2021 [49], where the antibody-mediated depletion was performed in 5×FAD animals in both cases, could be due to the different time windows used for treatment (2–3 months vs. 9–11 months, respectively). Indeed, the findings of Zhang et al., 2026 [54] suggest that antibody-mediated depletion in younger 5×FAD animals is consistent with the data reported by Feng et al., 2023 [50], further supporting the hypothesis that age plays a pivotal role in determining the function of B cells in AD. The removal of the spleen, the organ involved in B cells maturation [61], represents a different adaptive immune system-depleting method compared to genetic models-based. The divergence between Yu et al., 2022 [56] and Kim et al*.,* 2021 [49] might reside in the methodology used to study the role of adaptive immune system in AD (i.e., surgical intervention versus genetic deletion), albeit the APPswe/PSEN1dE9 animal model has been exploited in both studies. As demonstrated by Zhang et al., 2025 [53] and Zhang et al., 2026 [54] selecting a consistent intervention (i.e., adoptive transfer or precise genetic or molecular targeting of specific B cell subpopulations) could lead to more precise conclusions regarding the role of B lymphocytes in AD progression.

## The role of T cells in AD pathogenesis

T lymphocytes mature in the thymus from BM-derived cells and, after recognition of cognate antigens by T cell receptor (TCR), they undergo activation, clonal expansion, and differentiation to execute their effector functions [62]. These are CD3^+^ T cells, which can be subdivided into CD4^+^ and CD8^+^ T cells. The CD4^+^ T cells comprise of CD4^+^ helper and CD4^+^ regulatory T cells. Furthermore, T lymphocytes can also appear as γδ T cells and as double negative T cells. Although not officially categorized as a component of the adaptive immune system, mucosal-associated invariant T cells can recognize specific antigens through their TCRs.

There are contrasting results regarding the different proportion of peripheral blood T cells and their immunological reactivity in AD patients [63–68], nevertheless, the literature agrees that T lymphocytes are present in the CNS of AD patients (see for review [69, 70]).

### CD3^+^ T cells

Seminal studies revealed the accumulation of CD3^+^ T lymphocytes in different brain regions of postmortem specimen from patients affected by AD [71, 72], which were confirmed by more recent immunohistochemistry analysis [73–76]. Interestingly, the parenchymal infiltration of T cells was found to correlate with pTau load [74, 77]. Similar findings were observed in transgenic animal model of AD. Compared to age-matched WT animals, an increase in brain-infiltrated CD3^+^ T cells was observed in APPswe/PSEN1(L166P) animals from seven months of age onwards [78], as well as in APP^NL-G-F^ mice at different pathological stages [79, 80] and in 5×FAD mice at 6.5 months of age [81]. APP23 mice, an alternative AD animal model that carries a Swedish mutation and is characterized by parenchymal and vascular Aβ plaques [82], exhibited maximum CD3^+^ T cell accumulation in the brain at 20 months of age. This was accompanied by a propensity for attraction towards parenchymal plaques in comparison to vascular amyloid deposits [83]. Increased levels of brain infiltrated CD3^+^ T cells were first observed THYTau22 (encoding for human four-repeat tau mutated at sites G272V and P301S under a Thy1.2 promotor) [84], and subsequently in TauPS2APP mice (a triple transgenic mouse model combining tau and amyloid pathology) [85] and in a tauopathy mouse model carrying the APOE4 human allele, known to be associated with an increased risk of developing AD (P301S/human-APOE4-knock-in) [86].

Spatial transcriptomic analysis of 5×FAD brain revealed that cells neighboring T lymphocytes displayed higher levels of Major Histocompatibility Complex (MHC) Class II (MHC-II)-related and interferon (IFN)-stimulated genes, than non-neighboring cells. Furthermore, 18% of cells adjacent to T cells expressed the antigen presentation-associated marker CD74, compared to just 1% of neighboring cells around a randomly selected cell [40]. While these results indicated a possible involvement of brain-infiltrated T cells in AD within the context of classic adaptive immune responses, the direct correlation between the proximity of a cell presenting MHC-II and a T lymphocyte and the activation of an antigen-specific adaptive immune response remains to be substantiated. Furthermore, CD74 upregulation in this context may be indicative of activated microglial states rather than providing definitive evidence of functional antigen presentation [87, 88]. It is evident that further analysis is required to ascertain the classical role of T lymphocytes in adaptive immune responses in the AD brain. One potential avenue for this would be to validate the formation of functional immunological synapses.

To study the role of T cells in AD, researchers depleted T cells in different animal models of the disease (Table 3). Indeed, i.p. injections of anti-CD3 in 4-month-old THYTau22 mice reversed the spatial memory deficit and reduced the expression of neuroinflammatory markers in the hippocampus without affecting tau deposition or phosphorylation [84]. Similarly, nasal administration of anti-CD3 antibodies in 3×Tg-AD mouse model resulted in improved cognition in both the MWM and the Y-maze tests and reduced microglia activation (although with gender differences), without affecting Aβ pathology. Although the objective of the treatment was to increase T regulatory cells in the periphery, the authors observed an elevated rate of T cell migration to the brain. This finding suggests that there has been an anti-CD3 antibody-mediated expansion of peripheral T cells that have migrated to the brain to reduce microglial activation [89]. The findings of both studies indicated that, in addition to the divergent initial aims, the utilization of an anti-CD3 antibody could enhance cognitive function without exerting an effect on protein-aggregation-prone brain pathology. The same conclusion was reached using alternative strategies to prevent CD3^+^ T cells from infiltrating brain parenchyma. Treatment of 3×Tg-AD with fingolimod, which is known to increase the retention of T cells in lymphoid organs [93], had a positive effect on the working memory and brain pathology. This was characterized by a decrease in Iba1^+^ microglia cells, a reduction in pTau and APP expression levels, and a reduction of IL-6 together with a concomitant increase of IL-10 in the cortex [90]. However, it should be noted that a recent study performed on 9-month-old P301S transgenic mice i.p. injected with fingolimod, revealed an unexpected increase in the accumulation of CD4^+^ and CD8^+^ T cells in the hippocampal region, which was accompanied by a reduction in NeuN^+^ cell layers [91], raising questions about the response of adaptive immune cells to different administration routes (oral vs. i.p.). However, as was stated previously, experiments using fingolimod require careful interpretation. The targeting receptor (i.e. S1P) is expressed by several other cell types [45], which expands the possible mechanism of action and the potential mediators of its effects. Although both Lopes et al., 2023 [89] and Fagan et al., 2022 [90] observed a similar beneficial effect on the 3×Tg-AD mouse model, while anti-CD3-mediated depletion of T cells impacted cognition and neuroinflammation without modifying soluble Aβ oligomers [89], fingolimod-mediated restriction of brain infiltration by T cells reduced levels of pTau and APP [90]. This discrepancy may be due to the hypothesized mechanisms of action of the two treatments: anti-CD3 antibody intranasal delivery expanded peripheral T cells, which then migrated to the brain, whereas fingolimod trapped lymphocytes in the lymph node, preventing them from entering the CNS. This suggests that T cells entering the brain may not recognize, and therefore not react to, aggregation-prone proteins in the brains of 3×Tg-AD mice.

**Table 3:** Studies focusing on targeting T lymphocytes

Animal model	Intervention	Age of the intervention	Effects on the adaptive immune system	End of the experiment	Effects on AD-related pathology	References
THYTau22	i.p. injection of anti-CD3 (every two weeks)	4 months	↓ TCRβ^+^ T cells in blood, spleen and lymph nodes ↓ CD8^+^ T cells in the hippocampus	9 months	Beneficial: ↑ spatial memory in Y-maze task ↑ Arc and 14–3-3 protein expression ↓ *Clec7a*, I*tgax, Ccl3*, *Cd52*, *Gfap* and *Cd68* transcript levels No changes in Tau deposition and phosphorylation level	[84]
3×Tg-AD	intranasal delivery of anti-CD3 (three times week for five months)	1 month	↑ migration of T cells to the brain ↑ peripheral Tregs	6 months	Beneficial: ↑ cognition in the MWM and the Y-maze tests (in females only) No differences in Aβ pathology (intracellular Aβ and soluble Aβ oligomers) ↑ axiogenesis and synaptogenesis genes ↑ homeostatic microglia markers and ↓ inflammatory and antigen-presenting genes	[89]
3×Tg-AD	oral administration of Fingolimod (via drinking water for six months)	6 months	↓ circulating lymphocytes ↓ CD3^+^ cells in the cortex	12 months	Beneficial: ↑ memory deficits in the novel object location and the MWM tests ↓ Iba1^+^ microglia cells and ↓ reactivity (↑ branch number and length) ↓ IL-6 and ↑ IL-10 in the cortex ↓ pTau level in the cortex and the hippocampus ↓ APP expression level in the cortex	[90]
P301S	i.p. injection of Fingolimod	9 months	↓ CD19^+^ B cells, CD3^+^, CD4^+^, and CD8^+^ T cells in the blood ↑ CD4^+^ and CD8^+^ T cells in the brain	10 months	Detrimental: No effect on total tau levels Hippocampal shrinkage and lateral ventricle enlargement ↓ thickness of the NeuN^+^ cell layers	[91]
3×Tg-AD	i.p. injection of anti-TNFSF10 (twice a month)	3 months	↓ CD3^+^ cells in the hippocampus ↓ expression of T regulatory cells markers (G1TR and Foxp3) in the hippocampus	15 months	Beneficial: ↑ body and brain weight (and ↓ spleen weight) ↑ brain IL-10 ↓ microglial TNF-α levels ↓ Aβ_42_ and pTau levels	[76]
3×Tg-AD	i.p. injection of anti-TNFSF10 (twice a month for three months)	12 months	↓ PD1^+^CD4^+^ and CD8^+^ T cells in the spleen ↓ CD4^+^CD25^+^Foxp3^+^ T regulatory cells in both the brain and the spleen	15 months	Beneficial: ↓ Iba1 and CD86 and ↑ CD206 ↓ IL-1β, IL-2, TNF-α, IFN-γ, IL-12p70, IL-17A, IL-22, and IL-23 levels in the hippocampus ↓ brain-infiltrating proinflammatory monocytes (CD11b^+^LY6C^high^) ↓ Aβ plaques and pTau load	[92]
5×FAD	Crossbreeding with CD3e^-/-^	NA	Lacked both CD8^+^ and CD4^+^ T cells	3–12 months	Beneficial (but disease stage-dependent): ↓ Aβ plaques at early stage, but ↑ at later stage of the disease	[80]
APP^NL-G-F^	Crossbreeding with CD3e^-/-^	NA	Lacked both CD8^+^ and CD4^+^ T cells	3–12 months	Beneficial (but disease stage-dependent): ↓ Aβ plaques at early stage, but ↑ at later stage of the disease ↓ levels of soluble and insoluble Aβ_42_ (at 3 months) Unaltered Iba1^+^ microglial proportions, and loss of homeostatic gene and induction of AD-associated marker	[80]
P301S/human-APOE4-knock-in	i.p. injection of anti-CD4 and anti-CD8 antibodies (every five days)	6 months	↓ CD3^+^ and CD8^+^ cells in the hippocampus	9.5 months	Beneficial: ↑ short-term memory and exploratory behavior (Y-maze test), ↑ amygdala-dependent and hippocampal-dependent memory (freezing in response to an auditory and a contextual cue) and ↑ nest-building behavior ↑ brain atrophy ↓ Iba1^+^ microglia number, ↑ P2RY12^+^ and ↓ MHC-II^+^ and CD11c^+^ microglia ↓ pTau immunoreactivity in the hippocampus ↓ plasma Nf-L	[86]
**5×FAD**	Crossbreeding with TCRa ^-/-^	NA	Lacked both CD8^+^ and CD4^+^ T cells	4 months	Detrimental: ↓ cognitive memory function in the Y-maze test ↑ Aβ plaque deposits	[79]

The table includes animal models, interventions and the age at which these are implemented (where applicable, otherwise stated as not applicable, NA), the effects on the adaptive immune system, the age at which behavioral tests and sacrifice are performed, and the effects on AD-related pathology

Reducing CD3^+^ T cells immunoreactivity in the hippocampal region of 3×Tg-AD mice by targeting tumor necrosis factor ligand superfamily member 10 (TNFSF10), a pro-apoptotic cytokine which has previously been shown to have beneficial effects in this animal model [94], is accompanied by a reduction in levels of Aβ_42_, pTau, IL-1β, iNOS and microglial TNF-α [76]. Furthermore, the recent use of an anti-TNFSF10 antibody has been shown to alleviate AD pathology in 3×Tg-AD mice undergoing treatment. This treatment switched the phenotype of microglia towards an anti-inflammatory state, reducing amyloid plaque and pTau protein load [92]. A recent study taking advantage of genetically modified animals, knockout for the co-receptor CD3 (CD3e^-/-^), supported a detrimental role of T cells in AD pathology. T cell absence in APP^N-L-GF^ mice led to decreased levels of soluble and insoluble Aβ_42_ early stage of the disease. Remarkably, while at with early stage CD3 absence significantly reduced amyloid plaque burden, at later timepoints double transgenic mice (both APP^N-L-GF^/Cd3e^-/-^ and 5×FAD/CD3e^-/-^) showed increased plaque accumulation [80]. This suggests again a role for the adaptive immune system in AD pathology that depends on disease stage.

As for total CD3^+^ T cells, the number of both CD4^+^ and CD8^+^ T cells increase in the brain of 6-month-old APPswe/PSEN1(M146L) animals [95], and in spinal cord of P301S mice at 8 months of age [96]. When T cells were depleted using a combination of anti-CD4 and anti-CD8 antibodies in P301S/human-APOE4-knock-in mice, the researchers observed improvements in the Y-maze test and in the nest-building behavior, as well as amelioration of brain atrophy and reduction in microgliosis and decreased hippocampal pTau immunoreactivity and plasma neurofilament light chain (Nf-L) [86]. Although the above studies indicated that total CD3^+^ T lymphocytes have an adverse effect on AD pathology [84, 86, 89], one crossbreeding experiment involving 5×FAD and TCRa^-/-^ mice, which lack both CD4^+^ and CD8^+^ T cells, suggested that T lymphocytes may have a positive impact on AD pathology. Double transgenic mice exhibited impaired cognitive memory function and increased Aβ plaque deposits in the brain [79]. These results again highlight the differences between the modification of the adaptive immune system throughout life (i.e., using genetic-mediated techniques) and during life (i.e., using antibody-mediated treatment) in different AD animal models.

As none of these studies considered the role of different T cell subpopulations separately, a detailed analysis of different T cell populations is required to determine the role of T lymphocyte-mediated adaptive immunity in AD progression.

### CD4^+^ T cells

CD4 single-positive naïve T cells undergo the differentiation process via antigenic stimulation through the interaction of the TCR and the co-receptor CD4 with antigen-MHC-II complex, presented by professional antigen-presenting cells. Although different subsets of CD4^+^ T cells have specific functions in physiological and pathological condition, they are all involved in modulating the immune response through the secretion of specific cytokines [97].

Despite the enhanced clonal expansion and the reduced TCR diversity in the cerebrospinal fluid (CSF) of patients with AD, the frequency of CD4^+^ T cells is slightly lower than in healthy subjects [98]. A divergent scenario is observed within the parenchyma, wherein an augmented accumulation of CD4^+^ T lymphocytes is evident in the brains of patients afflicted with tau-associated AD pathology (specifically those diagnosed with frontotemporal lobar degeneration and Pick’s disease) [99]. Infiltration of these cells into the brain was also observed in animal models of both amyloidosis and tauopathy. Indeed, flow cytometry analysis revealed an increase in parenchymal CD4^+^ T cells in the brains of 6–7-month-old APP/PS1 mice [100], starting from 12 months of age in the brains of 5×FAD animals [101], and in the brains of 6-month-old 3×Tg-AD mice when compared to age-matched control animals [28]. As the CD4^+^ T cells activation depends on the antigen presentation via MHC-II, the decreased number of MHC-II^+^ cells and increased intracellular MHC-II expression in the microglia of APPswe/PSEN1dE9 mice, suggest an impaired antigen presentation process to CD4^+^ T lymphocytes in AD pathology [102, 103]. Notably, MHC-II^-/-^/5×FAD mice exhibited an exacerbated amyloid pathology in terms of a higher plaques load [104].

The role of CD4^+^ T cells in the brains of AD animal models has mainly been associated with detrimental effects (Table 4). I.v. injection of an anti-CD4 antibody improved cognitive performance in the Y-maze test in 5×FAD mice [51]. Increased extravasation of CD4^+^ T cells expressing the α4 integrin chain, CD49d, was observed near VCAM-1^+^ brain vessels in the brains of 6-month-old 3×Tg-AD mice. Anti-α4-integrin antibody treatment, which was suggested to halt the interaction of CD49d^+^CD4^+^ cells with the brain venule endothelium, improved the cognitive functions of 3×Tg-AD mice in the contextual fear conditioning (CFC) and the MWM tests. Neuropathological analysis of the treated mice revealed reduced microglial activation and decreased levels of Aβ and pTau [28].

**Table 4:** Studies focusing on targeting CD4^+^ T lymphocytes

Animal model	Intervention	Age of the intervention	Effects on the adaptive immune system	End of the experiment	Effects on AD-related pathology	References
5×FAD	i.v. injection of anti-CD4 antibodies	6.5 months	↓ CD4^+^ T cells in cortex	35 d.p.i	Beneficial: ↑ cognitive performance in the Y-maze test	[51]
5×FAD	s.c. administration of Xpro1595 (twice weekly for two months)	2 months and 4 months	↓ activated CD4^+^ T cells in the brain (and increase naïve T cells in the deep cervical lymph nodes)	4 months and 7 months	Beneficial: ↓ Aβ plaques in the subiculum ↓ cytokines expression (TGFβ and CCL2) ↑ LTP (at 4 months of age)	[105]
5×FAD	Oral gavage with an EP2/4 antagonist (for seven days)	4 months	↓ overactivation of spleen-isolated CD4^+^ T cell	8 d.p.i	Beneficial: ↓ Aβ plaques ↓ Iba1^+^ microglia	[106]
APPswe/PSEN1dE9 and 3×Tg-AD	Photobiomodulation therapy directed to the lymph nodes (ten minutes daily for one month)	6 months	↑ IFN-γ^+^ and IL-10^+^ CD4^+^ cells in the brain	7 months	Beneficial: ↑ spatial learning and memory abilities in the MWM test ↑ brain IGF-1 (only in APPswe/PSEN1dE9 mice), BDNF and TGFβ1 ↑ expression of IFN-γ and IL-10 ↓ GFAP^+^ astrocytes ↑ Iba1^+^ microglia cells	[107]
5×FAD	i.v. (retroorbital) adoptive transfer of CD4^+^ CAR-T recognizing fibrillar Aβ (twice, with a three-week interval)	6 months	↑ CD3^+^ and GFP^+^ cells at amyloid-rich arachnoid cuff exit points at the dural bridging veins ↑ T cells, CD4^+^ T cells and activated CD69^+^CD25^+^ cells in the brain tissue with leptomeninges	7.5 months	Beneficial: ↓ Aβ coverage in the dura but not in the parenchyma	[108]
5×FAD	i.v. (retroorbital) adoptive transfer of transiently nucleofected primary CD4^+^ T cells with mRNA encoding the CAR recognizing fibrillar Aβ (every ten days, over four weeks, for three total doses)	6 months	*NR*	7 months	Beneficial: ↓ total Aβ plaque load and Methoxy^+^ dense plaque cores ↓ Iba1^+^ and GFAP^+^ coverage no significant change in Lamp1^+^	[108]

The table includes animal models, interventions and the age at which these are implemented (where applicable), the effects on the adaptive immune system, the age at which behavioral tests and sacrifice are performed, and the effects on AD-related pathology. d.p.i: days post injection. *NR*: not reported

Despite the absence of any explicit focus on adaptive immune system modification, a few studies have demonstrated alterations in AD-associated pathologies in conjunction with modulation of CD4^+^ T cell activity. Administering Xpro1595, a compound capable of sequestering TNF-α, intraperitoneally was shown to reduce the number of infiltrating CD4^+^ T cells in the brains of 5×FAD animals. Treatment with Xpro1595 was also found to reduce the number of activated CD11b^+^ immune cells and decrease Aβ plaques, while also rescuing long-term potentiation (LTP) impairment [105]. In a similar manner, the oral gavage administration of a prostaglandin E2 synthetase antagonist has been demonstrated to reduce CD4^+^ T cell overactivation and result in a decrease of Aβ plaques and microgliosis in 5×FAD mice [106].

The detrimental effect of CD4^+^ T cells on AD brain was counteracted by photobiomodulation therapy, which was shown to enhance the recruitment of activated CD4^+^ T cells to the brain. Indeed, 3×Tg-AD- and APPswe/PSEN1dE9-treated mice showed reduced cognitive impairments in the MWM test, increased hippocampal neurogenesis and decreased astrocytosis [107]. However, it cannot be ruled out that the positive effects observed in this study are due to the photobiomodulation treatment itself, rather than to the activation status of the brain-infiltrating CD4^+^ T cells. Nevertheless, further experiments are needed to clarify the role of the different CD4^+^ T cell subsets in AD pathology. For example, a recent study demonstrated that the adoptive transfer of CD4^+^ effector memory T cells, which were found to be elevated in the blood of patients with mild cognitive impairment (MCI), into APP/PS1 mice exacerbated cognitive impairment and increased amyloid burden [109].

Whilst most of the aforementioned studies indicated that CD4^+^ T cells may be implicated in the progression of AD pathology, recent advancements in the field have led to the development of engineered CD4^+^ T cells with chimeric antigen receptors (CARs) that target the fibrillar forms of Aβ [108]. CARs are synthetic receptors leading to an MHC-independent activation of the TCR signaling [110]. The authors generated these CD4^+^ CAR-T cells in a hybridoma cell line in which green fluorescent protein (GFP) expression is controlled by the Nuclear Factor of Activated T-cells promoter. GFP is only expressed when the CAR successfully recognizes its specific antigen. Retroviral transduction of these cells was performed using a vector containing a linker-mediated fusion of the variable heavy and light chain regions (constituting the single-chain antibody fragment) of Lecanemab (a clinically-approved anti-amyloid antibody [111]), fused to the CD28 costimulatory region and an intracellular signaling region containing CD3ε with three immunoreceptor tyrosine-based activation motifs (ITAMs) (named Lec28z). Lec28z CD4^+^ CAR-T cells were adoptively transferred from wild-type littermates into 6-month-old 5×FAD animals and were found to be localized in the dura, where they reduced amyloidosis compared to mice that received PBS or control CD4^+^ CAR-T cells. To address the issue of prolonged T cell activation, which could potentially lead to an autoimmune response or non-specific cytotoxicity [112], primary CD4^+^ T cells were transfected with mRNA encoding the Lec28z receptor. Adoptive transfer of these cells, which transiently expressed CAR, resulted in a significant reduction in parenchymal plaque deposition and a decrease in microgliosis and astrogliosis in 5×FAD mice, compared to control animals injected with T cells that had been transfected with GFP mRNA [108]. These results provide a proof-of-concept for applying CAR-T based cellular immunotherapy to AD.

### CD4^+^ helper T cells

CD4^+^ helper T cells (Th) play a pivotal role in all aspects of the immune response. They can be subdivided into various subsets. Th1 cells protect against intracellular bacteria and viruses and are the main source of IFN-γ and TNF-α. Th2 cells secrete the interleukins IL-4, IL-5, and IL-13 and they facilitate tissue repair. They also play a significant role in humoral immunity and the organization of defenses against extrinsic parasites, and they significantly influence allergic reactions [113]. Th9 cells express IL-9 and are linked to allergic reactions, helminth host immunity, autoimmune disorders and inflammation [114]. Th17 cells express IL-17 and IL-22 and are important for protection against extracellular pathogens (mainly bacteria and fungi), as well as involved in inflammatory and autoimmune disorders. Th22 cells release IL-22, and are involved in tissue healing and host defense at barrier surfaces [115]. Follicular Th (Tfh) cells promote B cell proliferation and maturation, as well as modulating high-affinity antibody production [62].

To understand the role of Th cells in AD animal models, experiments were mainly performed via adoptive transfer using cells exposed to Aβ (Table 5). The first seminal adoptive transfer experiment using Aβ-specific mixed Th cells in APP/PS1(M146L) mice demonstrated an increased working memory abilities in the radial arm water maze (RAWM) test, alongside with fewer plaque-associated microglia and soluble Aβ. However, the cognitive and neuropathological improvements were not observed in animals receiving T cells enriched for the Th1 population, suggesting that a Th2-to-Th1 switch reduces the beneficial effects of the adoptive T cells transfer on AD pathology [116]. Similarly, the transfer of T cells, which were isolated from Aβ-vaccinated donors and re-stimulated in vitro to bias a Th2 profile, improved working memory of APP/PS1(M146L) mice in the RAWM test. The brains of the treated animals revealed a reduction in plaque-associated microglia cells and showed only reduced vascular amyloidosis, without any alteration to total Aβ plaque loads [117]. Th1 cells have also been demonstrated to exert a beneficial effect on AD pathology. Indeed, i.c.v. injection of Aβ-specific Th1 cells (from Aβ_42_-immunized mice) in APPswe/PSEN1(L166P) mice reduced Aβ plaques load and increased doublecortin (DCX)-positive neuronal progenitors [118]. Similarly, i.c.v. injection of Aβ-specific Th1 cells in 5×FAD mice reduced cortical Aβ plaque load, increased the number of brain MHC-II^+^ cells suggesting that Aβ-specific Th1 cells induce a protective immune response [104]. A reduction in Aβ plaque load has been also observed with Aβ-specific Th1 cells overexpressing BDNF. This beneficial effect was supported by the observation that the treated 5×FAD animals showed an increased NeuN protein levels in the hippocampus [119]. However, the protective effect of Th1 cells appears to depend on the route of administration. Indeed, the i.v. injection of Aβ-specific Th1 cells in 6-month-old APP/PS1 mice resulted in impaired cognitive function during the MWM test, alongside increased CD11b^+^ microglia and Aβ deposition [100]. Similarly, when both Aβ-reactive Th1 and Th17 cells were transferred via i.v. injection in APP/PS1(M146L) animals, the treated mice showed increased soluble Aβ_40_ and Aβ_42_ as well as decreased levels of DCX^+^ neuronal progenitor cells. However, only Th1 cells increased Aβ plaques load and microglial activation [120].

**Table 5:** Studies focusing on targeting CD4^+^ Th cells

Animal model	Intervention	Age of the intervention	Effects on the adaptive immune system	End of the experiment	Effects on AD-related pathology	References
APP/PS1(M164L)	i.v. injection of Aβ-specific mixed T cells and Th1 cell adoptive transfer	8 months	*NR*	14 d.p.i	Beneficial: ↑ working memory in the RAMW test ↓ Aβ plaque-associated microglia ↓ soluble Aβ NOTE: no changes observed in mice treated with Th1 cell alone	[116]
APP/PS1(M164L)	i.v. injection of Th2-biased Aβ-specific cells	11 months	*NR*	12.5 months	Beneficial: ↑ working memory in the RAMW test ↓ Aβ plaque-associated microglia Unaltered Aβ plaques (but ↓ vascular amyloidosis) ↓ plasma levels of IL-2, IL-4, GM-CSF, IFN-γ and TNF-α	[117]
APPswe/PSEN1(L166P)	i.c.v. injection of Aβ-specific Th1 cells	12–15 months	↑ Aβ-specific Th1 cells in the brain	28 d.p.i	Beneficial: ↓ Aβ plaques load No changes in apoptotic cell number ↑ proliferating neuronal progenitors (DCX^+^)	[118]
5×FAD	i.c.v. injection of Aβ-specific Th1 cells (secreting IFN-γ, from two-months-long Aβ_42_ immunized mice)	9 months	↑ MHC-II^+^ cells in the brain	11–21 d.p.i	Beneficial: ↓ cortical Aβ plaques ↑ brain *CD74*, *Infg*, *Cxcl9*, *Cxcl10*, *Cxcl11* transcript levels ↑ brain MHCII^+^ cells NOTE: These effects are lost in 5×FAD/MHCII^-/-^ mice	[104]
5×FAD	i.c.v. injection of Aβ-specific Th1 cells expressing BDNF	10–11 months	*NR*	10 d.p.i	Beneficial: ↓ Aβ plaque load in the cortex ↓ brain *Il1b* and *Sirp1b* transcript levels ↑ NeuN and BACE-1 protein levels	[119]
APP/PS1 (mutations not specified)	i.v. injection of Aβ-specific and Th1-polarized CD4^+^ T cells	6–7 months	↑ Aβ-specific Th1 cells in the brain	14 d.p.i	Detrimental: ↓ spatial learning in the MWM test ↑ activated microglia (CD11b^+^) in the cortex ↑ Aβ deposition NOTE: These effects are attenuated by anti–IFN-γ injection	[100]
APP/PS1(M164L)	i.v. transfer of Aβ-immunized Th1/17-polarized CD4^+^ T effector cells (twice at one-week intervals)	4–5 months	↑ CD4^+^ T cells reacting to an Aβ carrying MHC-II tetramer	35 d.p.i	Detrimental: slight ↓ spatial learning and memory in the RAWM test ↑ soluble Aβ_40_ and Aβ_42,_ and Aβ plaques (only in Th1-treated mice) ↑ reactive microglia (only in Th1-treated mice) ↓ neuronal progenitor cell (DCX^+^) cells ↓ PSD95 (only in Th1-treated mice) and SYP protein levels	[120]
5×FAD	i.p. injection of anti-CD38 (every second day for three weeks)	10–12 months	↓ meningeal IL-17^+^CD4^+^ T cells	11–13 months	Beneficial: ↑ physical and metabolic features in the Tensor Component Analysis Method ↑ working memory in the NOR test, but no effect on locomotor ability and anxiety-related parameters in the OF test No effect on Aβ plaques and soluble Aβ_42_ ↓ glucose level in the hippocampus ↑ genes involved in neuronal synaptic plasticity (*Homer1*), autophagy (*Mtor*) and learning and memory (*Igf2r*) ↓ IL-1β protein and transcript levels	[121]
3×Tg-AD	oral gavage with myricetin (for eight weeks and a half)	8 months	↓ proportion of RORγt^+^ Th17 cells in the peripheral blood and the hippocampus	10 months	Beneficial: ↑ spatial learning and memory in the NOR and the MWRM tests ↓ neuronal damage, with ↓ nuclear pyknosis and neuronal loss ↓ GFAP^+^ astrocytes and Iba1^+^ microglia ↓ Aβ plaques, APP levels and both cortical and serum Aβ levels ↓ pTau levels	[122]
5×FAD	i.v. injection of IL-21 (twice weekly for a total of five injections)	3 months	↑ Tfh cells and B plasma cells	3.5-4 months	Detrimental: ↑ Aβ plaques ↑ CD68 immunoreactivity ↑ IL-18 and IL-6 levels in the brain	[123]

The table includes animal models, interventions and the age at which these are implemented (where applicable), the effects on the adaptive immune system, the age at which behavioral tests and sacrifice are performed, and the effects on AD-related pathology. d.p.i: days post injection. *NR*: not reported

Data from an Aβ-injected rat model also supports the idea that Th17 cells are detrimental, as transferring these cells causes damage to synaptic plasticity by triggering the release of proinflammatory factors such as FasL, IL-17 and IL-22 [124]. Recently, injecting an anti-CD38 antibody to reduce the number of meningeal IL-17^+^ CD4^+^ T cells was found to improve physical and metabolic features, as well as recognition memory in the NOR test, in 5×FAD mice. This effect was accompanied by a reduction in IL-1β, with no impact on Aβ pathology. The authors hypothesized that this antibody-mediated effect may be associated with the inhibition of IL-17A/IL-1β signaling between the meninges and the brain [121]. An increased number of IL-17A-producing CD4^+^ T cells was recently observed in the superficial cervical lymph nodes of 12-month-old male 3×Tg-AD mice compared to wild-type mice [125]. This suggests that the infiltration of Th17 cells into the AD brain parenchyma may be harmful. A recent study on the effects of myricetin, an inhibitor of the transcription factor RORγt involved in Th17 polarization, provided support for the idea that Th17 cells have a detrimental effect. Treatment with myricetin improved the cognitive function of 3×Tg-AD mice in the NOR and the MWM tests. Treatment also reduced neuronal damage and decreased levels of Aβ plaque and pTau, while attenuating glial activation [122].

The role of Tfh cells in AD is not well understood, except for their suggested involvement in the secretion of IL-21, which is found at increased levels in the plasma of AD and MCI patients compared to age-matched controls. Although further experiments are required, IL-21 administration in 5×FAD mice resulted in increased splenic Tfh cells, and an increase in Aβ deposition and in CD68 immunoreactive microglia. These results suggest that Tfh cells may play a detrimental role in AD progression [123].

In conclusion, while the i.c.v. administration of Th1 cells appears to be effective in reducing AD pathology as suggested by Fisher et al., 2014 [118], Mittal et al., 2019 [104] and Eremenko et al., 2019 [119], the i.v. administration is associated with reduced or detrimental effects, as observed by Ethell et al., 2006 [116], Browne et al., 2013 [100] and Machhi et al., 2021 [120]. The adoptively transferred Aβ-specific Th2 cells used by Cao et al., 2009 [117] seem to exert a beneficial effect without infiltrating the CNS, raising questions about their potential modes of action. Further experiments on Th2 transfer are required to determine whether they could potentially affect the brain borders. While the effect of Th17 cells on worsening AD pathology, as observed by Zhang et al., 2013 [124], Peralta Ramos et al., 2025 [121] and Li et al., 2025 [122] is well established, the role of Tfh cells requires further investigation to determine whether they have a detrimental impact on AD progression, as suggested by Agrawal et al., 2022 [123].

### CD4^+^ regulatory T cells

Regulatory T cells (Tregs) are characterized by the transcription factor Foxp3 and the expression of CD25 marker. In physiological environments, they have the function to maintain immune tolerance by suppressing the activation and proliferation of other immune cells [62]. However, it has been suggested that the activity of regulatory cells is decreased during AD-related inflammatory conditions, thereby worsening the pro-inflammatory environment [126]. Flow cytometry analysis of APP/PS1 brain tissue showed that Tregs decreased or remained unchanged in the early stages of the disease, but increased later during disease progression, which was characterized by increased inflammation [127–129].

Cell depletion studies support the beneficial role of Tregs in AD pathology (Table 6). Depleting Tregs in 3×Tg-AD mice, using anti-CD25 antibody, resulted in marked spatial learning deficits in the MWM test, as well as increased Aβ plaque deposition, decreased glucose metabolism and increased microgliosis [130]. Similarly, anti-CD25 treatment resulted in earlier cognitive impairment in the Barnes maze test and reduced the number of plaque-associated microglia in APPswe/PSEN1(L166P) mice, without affecting Aβ deposition or overall microgliosis [131]. This suggests that the antibody-mediated depletion of Tregs has detrimental effects on cognition that bypass Aβ pathology. A follow-up study revealed that, while late Treg depletion via anti-CD25 does not affect astrocyte reactivity or morphology associated with plaques, early intervention modulates astrocyte phenotypes. This is demonstrated by increased expression of the A1-like astrocyte marker C3 and decreased expression of the A2-like astrocyte marker cyclooxygenase 2 (COX-2) [134]. These results imply that Tregs play a time-dependent role in regulating reactive astrocytes in AD-like pathology.

**Table 6:** Studies focusing on targeting CD4^+^ Tregs

Animal model	Intervention	Age of treatment	Effects on the adaptive immune system	End of experiments	Effects on AD-related pathology	References
5×FAD	i.p. injection of anti-CD25 (once a week for one, three and five months)	1 month	↓ Tregs	1, 3 and 6 months	Detrimental: ↓ spatial learning memory in the MWM test ↑ Aβ plaques deposition (only in the 5-months-treated group) ↓ glucose metabolism ↑ microglia cells (only in the 5-month-treated group) NOTE: no changes after one month treatment	[130]
APPswe/PSEN1 (L166P)	i.p. injection of anti-CD25 (twice every month)	1 month	Transient ↓ of Tregs (CD4^+^CD25^+^Foxp3^+^)	4, 5 and 7 months	Detrimental: ↓ learning functions in the Barnes maze test Unaltered Aβ pathology ↓ plaque-associated microglia cells (Iba1^+^CD68^+^), without affecting total microglia cells number ↑ number of C3^+^ and ↓ COX2^+^GFAP^+^ astrocytes	[130, 131, 134]
3×Tg-AD	i.p. injection of bee venom phospholipase A2 (once a week for three months)	3 months	↑ Treg population in the spleen but ↓ CD4^+^ T cell infiltration in the brain	6 months	Beneficial: ↑ spatial learning deficits in the MWM test ↓ Aβ deposits ↑ glucose uptake in the brain ↓ CD11b^+^ microglia	[132]
APPswe/PSEN1dE9	i.p. injection of AAV8-IL-2	8 months	↑ systemic and brain Tregs (CD4^+^CD25^+^)	13.5 months	Beneficial: ↑ spatial memory in the MWM task ↑ LTP-related synaptic plasticity and ↑ spine density ↓ Aβ_42_/Aβ_40_ ratio and hippocampal Aβ plaque levels (and ↑ APP levels) ↑ amyloid plaques-associated astrocytes	[133]
APPswe/PSEN1dE9	i.p. treatment with recombinant IL-2 (every other day, for three months)	6 months	↑ Tregs in the spleen and the brain	9 months	Beneficial: ↑ cognitive function in the MWM test ↑ brain and serum level of TGF-β, IL-17A and IL-22, serum level of IL-10, and brain level of TNF-α and IL-1β ↓ cortical Aβ plaques load ↓ insoluble Aβ_42_ (no changes in the soluble form) ↓ resident (CD45^int^CD11b^+^CD11c-) and activated microglia (CD68^+^), but ↑ plaques associated CD68^+^ microglia	[128]
APPswe/PSEN1 (L166P)	i.p. administration of IL-2 (daily treatments for five days repeated every three weeks)	1.5 months	↑ Tregs (CD4^+^CD25^+^Foxp3^+^)	4, 5 and 7 months	Beneficial: ↑ learning capacity in the Barnes maze test Unaltered Aβ pathology ↑ plaque-associated microglia cells (Iba1^+^CD68^+^) Unaltered astrocyte morphology, number and phenotype	[131, 134]
APPswe/PSEN1dE9	intracardiac injection (twice, with one week apart) with CD4^+^CD25^+^ Tregs (educated with human umbilical cord derived mesenchymal stem cells)	6 months	↑ Tregs	7 months	Beneficial: ↑ learning and memory abilities in the MWM test ↓ Aβ plaques and ↓ soluble Aβ_40_ and Aβ_42_ ↓ Iba1^+^ microglia	[135]
3×Tg-AD	i.v. injection of Tregs (CD4^+^CD25^+^)	4 months	*NR*	6 months	Beneficial: ↑ learning and memory in the MWM test ↓ brain IL-6, IFN-γ and IL-17A levels ↓ Aβ plaques burden in the cortex ↓ Iba1^+^ microglia	[130]
3×Tg-AD	i.v. adoptive transfer of CX3CR1^+^ Tregs	5 months	↑ suppressive capacity of Tregs	14 d.p.i	Beneficial: ↑ spatial working memory in the passive avoidance and the Y-maze tests ↓ Aβ accumulation ↓ Iba1^+^ immunoreactivity ↓ brain inflammatory response (*Cx3Cr1*, *Csf1*, *Cd86*, *iIos*, *Il1b and Il12a* at transcript level, and TNF-α, IL-1β, IL-6 and CD86 at protein levels)	[136]
3×Tg-AD	i.v. infusion of Aβ antigen-specific Tregs (CD4^+^CD25^+^)	4 months	*NR*	6 months	Beneficial: ↑ cognitive function in the Y-maze and the passive avoidance tests ↓ Aβ accumulation No alterations in brain glucose metabolism ↓ Iba1 protein levels and shift from pro- to anti-inflammatory signal of microglia ↑ MBP protein levels No changes in GFAP^+^ signal	[137]
3×Tg-AD	i.v. infusion of human CD4^+^CD25^+^Foxp3^+^ Tregs isolated from human peripheral blood, exposed to Aβ peptide	10 months	*NR*	14 months	Beneficial: ↑ spontaneous alternation in the Y-maze test ↑ escape latency in the passive avoidance test ↓ brain expression level of *Nos2*, *Cd86*, *Il1b*, *Tnf*, *Trem2*, *Itgax* and *Csf1* transcripts No changes in TREM2^+^CD11b^+^ microglia cell numbers	[138]
3×Tg-AD	Intrathecal injection of human CD4^+^CD25^+^Foxp3^+^ Tregs isolated from human peripheral blood, exposed to Aβ peptide (Aβ-hTregs, either 1 × 10^3^ or 1 × 10^4^ cells)	10 months	*NR* NOTE: Detection of Aβ-hTregs in the spinal cord and the brain from four hour up to thirty days after the infusion (experiment performed in BALB/c nude mice)	12 months	Beneficial: ↑ spontaneous alternation in the Y-maze (only in mice receiving 1 × 10^3^ cells) ↑ escape latency in the passive avoidance test (only in mice receiving 1 × 10^3^ cells) ↓ brain expression level of *Nos2*, *Cd86*, *Il1b*, *Tnf, Il12*, *Il23*, *Il6*, *Trem2*, *Tmem119*, *Cx3cr1, Itgax* (only in mice receiving 1 × 10^3^ cells) and *Ccl6* transcripts ↓ Iba1^+^Nos2^+^ microglia ↓ hippocampal Aβ and pTau levels	[138]
APP/PS1(M164L)	i.v. injection of Tregs expressing an Aβ-specific TCR (three times once a week)	8 months	↑ brain Tregs (CD4^+^CD25^+^FoxP3^+^) and ↑ suppressive function of splenic Tregs after Aβ treatment in vitro	28 d.p.i	Beneficial: ↑ memory function in the RAWM test (no changes in the Y-maze test) ↑ brain glucose uptake ↓ soluble and insoluble Aβ peptides, and dense amyloid plaque deposition in both the cortex and the hippocampus ↓ numbers of Iba1^+^ microglia in the cortex ↓ cortical levels of *Trem2* and *Gfap* transcripts	[139]
5×FAD	i.v. injection of Aβ-specific Tregs (CD4^+^CD25^+^)	2–3 months	*NR*	4–5 months	Beneficial: ↑ cognitive function in the Y-maze and the passive avoidance tests ↓ Aβ levels in the hippocampal CA1 region ↓ pTau signal ↓Nos2 deposition ↓ pro-inflammatory markers (*Nos2*, *Tnfa*, *Il1b*, and *Il6*) ↑ anti-inflammatory markers (*Arg1* and *Mrc1*)	[140]
APP/PS1(M164L)	i.v. transfer of nanoparticle enriched Aβ_42_-specific CD4^+^ T cells (twice over two weeks)	6 months	↑ brain Tregs (Foxp3^+^)	14 d.p.i	Beneficial: ↑ exploration time in the NOR test (but no changes in the MWM test) ↓ Aβ burdens in both the hippocampus and cortex, ↓ soluble and insoluble Aβ_42_ and Aβ_40_ (but no effect on Aβ_42_ in the CSF) ↑ Iba1^+^ cells and↑ Aβ uptake by microglia ↑ expression of anti-inflammatory marker (IL-10 and TGF-β) and ↓ pro-inflammatory cytokines (IL-1β, TNF-a and IL-6) ↑ SYP and PSD95 protein levels in the brain ↑ *Pdcd1*, *Msr1*, *Clec7a* and *Arg1*, and induction of several hallmark phagocytosis genes and inflammatory response genes (in sorted CD11b^+^)	[95]
APPswe/PSEN1dE9	intramuscular injections of influenza vaccine (every four weeks over the course of five months)	3 months	↓ CD4^+^Foxp3^+^ Tregs activity in spleen	8 months	Beneficial: ↑ cognitive deficits in the MWM and the OTF tests ↓ cerebral Aβ plaque burden and soluble and insoluble Aβ_40_ and Aβ_42_ ↑ microglia cells and enhanced microglial phagocytosis ↓ anti-inflammatory and increase pro-inflammatory cytokine levels NOTE: The phenotype was rescued with ATRA injections	[141]
5×FAD	i.p. injection of ATRA (every other day for one week)	5 months	↑ Foxp3^+^CD25^+^ Tregs in the spleen	6 months	Detrimental: ↓ spatial memory functions in the RAWM test ↑ Aβ plaques burden and soluble Aβ_40_ and Aβ_42_ ↑ GFAP^+^ astrogliosis	[142]

The table includes animal models, interventions and the age at which these are implemented (where applicable), the effects on the adaptive immune system, the age at which behavioral tests and sacrifice are performed, and the effects on AD-related pathology. d.p.i: days post injection. *NR*: not reported

The positive role played by Tregs in AD has also been demonstrated by the expansion of their pool through drug treatment. For example, treatment with phospholipase A2 from bee venom, which is known to increase the number of Tregs [143], has been shown to reverse spatial learning deficits in the MWM test and to decrease Aβ deposits, reduce microglia activation and increase glucose uptake in the brains of 3×Tg-AD mice [132]. As previously stated, the expansion of peripheral Tregs was also observed after the nasal administration of an anti-CD3 antibody in 3×Tg-AD mouse model, resulting in a beneficial effect on AD-like cognitive dysfunction [89]. An improvement in spatial learning has been observed in APPswe/PSEN1(L166P) mice following the expansion of the Tregs pool via IL-2 administration. However, these cognitive effects are not linked to changes in Aβ pathology or astrocytosis, but only to a raise in the number of plaque-associated microglia (Iba1^+^Cd68^+^) [131]. Similarly, inducing Treg expansion via an AAV8-IL-2 injection restored spatial memory deficits in APPswe/PSEN1dE9 mice in the MWM task. However, this treatment decreased the Aβ_42_/Aβ_40_ ratio and the number of Aβ plaques in the hippocampus, thereby promoting the recruitment of astrocytes around the plaques. Furthermore, it improved synaptic plasticity and prevented a reduction in spine density [133]. Expansion of Tregs in the spleen and brain following low-dose recombinant IL-2 i.p. treatment improved cognitive function also in the APPswe/PSEN1dE9 animal model. Treated mice revealed a reduced load of cortical Aβ plaques, reduced numbers of both resident (CD45^int^CD11b^+^CD11c^-^) and activated (CD68^+^) microglia, lowered the infiltration of monocytes (CD45^high^CD11b^+^CD11c^-^) and reduced the levels of TNF-α and IL-1β in the brain [128]. A recent phase II clinical trials involving the administration of low-dose IL-2 every four weeks in AD patients showed a decrease in inflammatory mediators in the blood and positive trends in clinical endpoints, due to a robust and sustained increase in the Treg population [144].

The protective role of Tregs was also supported by adoptive transfer experiments. Indeed, the intracardiac injection of CD4^+^CD25^+^ Tregs, which had been educated with human umbilical cord-derived mesenchymal stem cells to enhance their suppressive functions, improved the learning and memory capacity of APPswe/PSEN1dE9 mice. The adoptively transferred mice also exhibited reduced accumulation of Aβ plaques and decreased microgliosis [135]. Similarly, injecting Tregs into 3×Tg-AD mice decreased learning and memory deficits in the MWM test, reducing levels of IL-6, IFN-γ and IL-17A, as well as reducing the burden of Aβ plaques and the number of Iba1^+^ microglia cells in the cortical region. To demonstrate that the beneficial effect on AD pathology was limited to Tregs, parallel experiments involving CD25^-^ effector T cells were performed. This had no impact on Aβ plaque load or behavioral performance in the MWM test [130]. Moreover, the i.v. administration of ex vivo-derived human Tregs (CD4^+^CD25^high^) in 5×FAD/Rag2^-/-^/IL2ry^-/-^ mice reduced amyloid burden and lowered the number of GFAP^+^ reactive astrocytes and CD68^+^ microglial cells. It also downregulated pro-inflammatory cytokines, the complement cascade, and other markers of microglial activation [37]. Although the authors of this study relied on a xenogeneic model based on human Tregs that were adoptively transferred into AD mice, which represents a significant bias with respect to Treg immunobiology, the results suggested that the beneficial role of Tregs may be mediated by modulation of inflammation. More recently, experiments performed on 5-month-old 3×Tg-AD (Thy1.2^+^) mice undergone the adoptive transfer of CX3CR1^+^Thy1.1^+^-expressing Tregs, to encourage their migration to the brain, supported the protective role of Tregs in AD pathology. Compared to animals receiving control cells, these mice demonstrated improved spatial working memory function in the Y-maze and the passive avoidance tests. In terms of neuropathological outcomes, CX3CR1^+^ Tregs transfer reduced Aβ plaques and Iba1 immunoreactivity, as well as inflammatory responses in the brain, including TNF-α, IL-6, and IL-1β [136].

Experiments proving the protective role of Tregs were performed also using the adoptive transfer of Aβ-stimulated cells. The i.v. injection of Aβ-specific Tregs (CD4^+^CD25^+^ T cells isolated from the splenocytes of pre-existing Tregs-depleted mice that were immunized with aggregated Aβ) into 3×Tg-AD mice ameliorated cognitive impairment in the MWM, Y-maze and passive avoidance tests. It also decreased Aβ accumulation, Iba1^+^ signals and the related neuroinflammatory phenotype [137]. Similarly, i.v. injection of Aβ-specific Tregs in 5×FAD mice improved alternation scores in Y-maze and increased passive avoidance test escape latency. Neuropathologically, the treated mice showed decreased Aβ levels in the hippocampal region and reduced pTau intensity. The treatment revealed also a reduction in Nos2 protein deposition and decreased pro-inflammatory markers, as well as an increase in anti-inflammatory markers [140]. To find a potential allogeneic clinical therapy for patients with AD, CD4^+^CD25^+^Foxp3^+^ Tregs isolated from human peripheral blood were exposed to Aβ peptide in vitro (Aβ-hTregs) and then transferred to 10-month-old 3×Tg-AD mice. Four months after injection, the mice exhibited improved spatial working memory and exploratory behavior, as assessed by the Y-maze test, and reduced learning and memory impairments, as determined by the passive avoidance test. Gene expression analysis of the brain revealed reduced expression of inflammatory and disease-associated microglia markers. These findings were also confirmed following an intrathecal injection of a lower dose of cells, which was accompanied by a reduction in Aβ and pTau levels in the hippocampus [138]. Furthermore, a recent study on 6-month-old APP/PS1(M146L) mice, which had undergone adoptive transfer of Aβ_42_-specific CD4^+^ T cells that acquired a Treg phenotype in the brain, showed improved cognitive function in the NOR test. Brain pathology revealed a reduction in Aβ plaques load in both the hippocampus and cortex, a shift from a pro- to an anti-inflammatory cytokine environment and increased in expression of SYP and PSD95. Remarkably, the authors observed an increase in Iba1^+^ microglia associated with Aβ plaques, which, together with the induction of several phagocytosis and inflammatory response genes, and the abolishment of the neuroprotective effects with PLX5622 treatment, suggested the beneficial effect of Tregs dependent on microglia [95].

A beneficial effect was also observed when Tregs expressing a specific TCR that recognizes an Aβ sequence identified in Machhi et al., 2021 [120] into APP/PS1(M146L) mice, which resulted in increased glucose uptake in the brain compared to animals treated with the control. Neuropathological analysis revealed a reduction in insoluble Aβ and dense amyloid plaque deposition, as well as a reduced number of reactive Iba1^+^ cells in the cortex [139].

All the above studies suggested a protective role for Tregs in AD, opening the possibility of refining established therapies that target specific regulatory T cell subsets. Indeed, a recent Phase I clinical trial demonstrated the safety and tolerability of Aβ-hTregs (CD4^+^CD25^hi^CD127^lo^ cells isolated and expanded with IL-2 and Aβ) in patients with AD, although there were no significant improvements in exploratory efficacy assessments of cognitive function [138]. Another phase I clinical trial investigating the impact of administering Tregs to patients with AD is currently in progress (NCT03865017, for review see [145]).

However, few studies suggest a potentially detrimental effect of Tregs in AD. For example, the positive effects observed in Di Benedetto et al., 2019 [76] and Cantone et al., 2025 [92] were also associated with a reduction in Tregs in the brain. This suggests that, despite the well-established protective role of Tregs in AD, anti-TNFSF10 antibody-mediated modulation of these cells may have a detrimental effect on the disease. Furthermore, the indirect inhibition of peripheral CD4^+^Foxp3^+^ T cells via influenza vaccination improved cognitive deficits in APPswe/PSEN1dE9 mice and reduced amyloid plaque pathology by stimulating microglia phagocytosis and increasing the inflammatory response in the brain. Enhancing immunosuppression with all-trans retinoic acid (ATRA)—which stabilizes the Treg phenotype and increases the suppressive capacity of Tregs [146]—prevented influenza vaccine-induced protection and supported a potential beneficial effect of Tregs on AD pathology that is dependent on dosage [141]. However, treatment with ATRA exacerbated spatial memory deficits and increased the burden of Aβ plaques and gliosis in 5×FAD mice [142]. Therefore, results from Yang et al., 2020 [141] and from Baruch et al., 2015 [142] suggest that ATRA-mediated immunosuppression is only efficient when AD pathology occurs alongside an ongoing adaptive immune response (e.g., flu vaccine). This indicates that Tregs-mediated immunosuppression must be precisely regulated to avoid adverse effects on AD pathology. Nevertheless, in the near future, CAR Treg therapies have the potential to be impactful in the treatment of AD [147], as preliminary in vitro tests with CAR Tregs targeting tau and Aβ are present [148, 149], as well as aforementioned promising preclinical evidence for CD4^+^ CAR T [108] and CAR Treg-like approaches [80, 139].

### CD8^+^ T cells

Cytotoxic T lymphocytes that express the CD8 co-receptor can recognize cells presenting a specific antigen via MHC-I using their TCR. Once activated, these CD8^+^ T cells secrete granzymes (GZM) and perforin, which can induce apoptosis and destroy target cells. CD8^+^ T cells are the most prevalent type of T cell in several inflammatory and degenerative CNS diseases, such as MS, as well as viral- and tumor-related pathologies [150].

A significant increase in the number of parenchymal CD8^+^ T cells has been observed in both elderly individuals and in patients with neurodegenerative diseases [151–154]. The first report of CD8^+^ T cells in autopsy tissue of AD patients revealed their accumulation in brain capillaries [155]. More recently, various studies have demonstrated the presence of CD8^+^ T lymphocytes in the brain parenchyma of AD patients [73, 74, 101, 156–158]. However, recent single-nucleus RNA sequencing (snRNA-seq) data analysis from two distinct post-mortem AD cohorts revealed that there was no significant increase in CD8^+^ T cell frequency observed in individuals with pathologically confirmed AD in either the prefrontal cortex or the hippocampal region, but only in the middle temporal gyrus. While these results are based solely on snRNA-seq data, they suggest that CD8^+^ T lymphocytes enrichment in AD may be regionally restricted [159].

Researchers have demonstrated that CD8^+^ T cells begin to infiltrate the brain parenchyma from around 12–13 months of age and increase in number by 18–19 months in APPswe/PSEN1dE9 mice [156]. In 5×FAD mice, the parenchymal appearance of CD8^+^ T cells increased as early as 4 months of age compared to WT animals [40, 79, 101, 105, 160]. Furthermore, an immunohistochemical analysis revealed an accumulation of CD8^+^ T cells in the hippocampus of THY-Tau22 mice from 7 months of age [84].

A transcriptomic profile of brain-derived CD8^+^ T cells in APPswe/PSEN1dE9 animals revealed a tissue-resident memory (T_RM_) molecular identity, which was confirmed with immunohistochemistry and flow cytometry analysis [161]. A similar phenotype characterized by expression of the homing marker CXCR6 together with PD-1, CD69 and CD103 was observed in 5×FAD animals [79]. Furthermore, both flow cytometer analysis and immune cell-enriched scRNA-seq of brain tissue from 5×FAD mice revealed that CD8^+^ T_RM_ is the most prevalent T cell type and demonstrated increased levels of T cell exhaustion markers [101, 162]. Similar detailed scRNA-seq and flow cytometry analyses of the brains of 3×Tg-AD mice suggested an increased abundance of CD103^-^CD8^+^ T_RM_ cells, characterized also by the expression of GZMK. This increase in the abundance of CD103^-^CD8^+^GZMK^+^ T_RM_ was confirmed in the CSF and the hippocampus of AD patients [163]. Remarkably, analysis of spinal cord tissue from P301S mice has revealed that the majority of CD8^+^ T cells express GZMK [96]. Furthermore, a recent study has indicated GZMK to be a secreted pro-aging CD8^+^ T cell-derived factor. Inhibition of this serine protease has been demonstrated to result in improved cognition (using the RAWM test) in aged animals [164]. This suggests that it may serve as a viable therapeutic target for age-related diseases, including AD. However, other animal models of amyloidosis revealed a different profile for brain-resident CD8^+^ T cells. Flow cytometry analysis of these cells in 3-month-old APP^NL-G-F^ mice showed an increased PD-1 and reduced CD69, CD103 and CXCR6, findings that are consistent with those observed in 5×FAD animals analyzed in Su et al., 2023 [79]. These results highlight a significant skewing toward an effector phenotype, characterized by elevated frequencies of CX3CR1, CD4, and KLRG1 [80]. Similarly, a recent spectral flow cytometric analysis of aged APPswe/PSEN1dE9 mice (22–28-month-old) revealed an increased number of infiltrating CD8^+^ effector T cells that express IFN-γ, GZMB, and perforin [165]. Although the above-mentioned data may reflect differences in the profile of stage-specific CD8^+^ T cells in the various AD transgenic animal models, the T_RM_-like (CD69^+^CD103^+^) phenotype of CD8^+^ T cells has been confirmed in the CSF of AD patients using flow cytometry and scRNA-seq [166].

The phenotypic analysis of CD8^+^ T cells in the CSF of patients with AD revealed the presence of a specific subpopulation of effector memory T cells that re-express CD45RA (TEMRA) [68, 157], which are terminally differentiated T cells associated with protracted antigen exposure [167]. Recent pseudo-temporal trajectory analysis of blood and CSF single-cell sequencing datasets suggests that these CSF-resident CD8^+^ TEMRA cells may originate from blood-derived CD8^+^ T cells expressing TCF7, a transcription factor associated with cellular stemness, and GZMK [168], in line with the data reported in the study by Terrabuio et al., 2025 [163].

Recent analysis of APP23 animal brains revealed a particular subset of CD8^+^ T cells that characterized animals at the late stage of disease (24 months old), with elevated expression of interferon-stimulated genes (ISGs) [83]. Further spatial transcriptomics analysis indicated that T cells exhibiting an ISG signature were present in higher numbers in the plaque neighborhood, which is in line with the transcriptomic analysis of brain-isolated CD8^+^ T cells in aged APPswe/PSEN1dE9 animals observed by Altendorfer et al., 2022 [161]. These cells were found to be the source of the release of the chemokine, CXCL10, which is known to mediate CD8^+^ T cells infiltration in white matter degeneration [169] and to attract CXCR3-positive CD8^+^ T cells in AD [170]. This finding is strongly supported by the spatial analysis of frozen post-mortem tissue from patients with AD and suggests the presence of a local adaptive immune system-driven neuroinflammatory responses to amyloid deposits [83].

In terms of immunological function, flow cytometry analysis of ex vivo-isolated CD8^+^ T cells from aged ArcAβ mice (which overexpresses the human APP with both the Swedish and the E693G mutations) revealed reduced proliferative activity and IFN-γ production [102]. However, stimulation of CD8^+^ T cells isolated from the brains of 5×FAD mice using phorbol-12-myristate-13-acetate increased IFN-γ expression [101]. These results imply that variations in the phenotype of brain CD8^+^ T cells are not only specific to the mouse model, but probably also to age.

Once inside CNS structures, CD8^+^ T cells establish strong connections with other brain cell populations. CD8^+^ T cells have been observed near myelinated neuronal structures, such as axons and dendrites, in both human and mouse brain specimens [156, 157]. Furthermore, recent studies have suggested a bidirectional association between CD8^+^ T lymphocytes and microglia cells [40, 79, 101]. Indeed, while depleting microglia led to an increase in the number of CD8^+^ T cells in the brains of APPswe/PSEN1dE9 mice, a slight increased number of microglia populated the brain of CD8^+^ cell-depleted APPswe/PSEN1dE9 mice [156, 171]. However, a recent detailed multiplex immunohistochemistry analysis of microglia cells in brain tissue samples from AD patients suggested no direct association between CD8^+^ T cells and microglia [172]. The suggested microglia-mediated route responsible for CD8^+^ T cell infiltration [86, 173] (see for review [174]) may occur via different chemokine-chemokine receptor axes, including the CXCL16–CXCR6 axis [79, 175], the CXCL10–CXCR3 axis [170] or the CCL5–CCR5 axis [80]). In addition to the aforementioned, albeit still contested, hypothesis concerning alternative brain homing mechanisms, further proposals have been advanced. Indeed, the observation of fewer brain CD8^+^ T cells (particularly the pro-inflammatory and cytolytic subsets) in the brains of 5×FAD/J_H_T mice has suggested a B cell-mediated infiltration mechanism [101], indicating an intertwined communication among different cells of the adaptive immune system during AD pathology. Recent mechanistic analyses using knockout animals have revealed that the Leukocyte Function-Associated Antigen-1 L integrin may play a role in the accumulation of CD103^-^CD8^+^ T_RM_ cells in the brains of 3×Tg-AD mice [163]. However, the antigen-mediated mechanism is considered the most likely cause of CD8^+^ T cell infiltration in the brains of AD subjects. Indeed, analysis of immune cells in human CSF samples revealed that the proportion of activated Human Leukocyte Antigen-DR^+^CD8^+^ T cells was higher in patients with mild AD than in healthy elderly individuals. The authors also observed a significant correlation between CD8^+^ T cell antigen-dependent activation and microstructural tissue damage in the hippocampus [176].

CSF analysis based on scRNA- and TCR-seq revealed the presence of clonally expanded CD8^+^ T cells in the CSF of AD patients, indicating that an antigen-specific response is driving the expansion of these cells in AD [157]. In contrast, a subsequent TCR repertoire sequencing analysis revealed a significant increase in clonal expansion and a substantial reduction in TCRβ diversity in CSF-derived CD4^+^ T cells, but not CD8^+^ T cells, in individuals with AD compared with control subjects [98]. The discrepancy between Gate et al., 2020 [157] and Joshi et al., 2022 [98] may be due to differences in cell preparation (i.e., freeze-thawed versus fresh), technical (e.g., scRNA-seq versus genomic DNA analysis) and analytical factors (e.g., different calculations to define clonal expansion). Similarly, single cell analyses conducted on postmortem leptomeninges tissue samples indicated that the majority of expanded CD8^+^ T cells clonotypes exhibited a consistent reduction in size in AD compared to non-neurodegenerative control subjects, suggesting an increase in CD8^+^ T cells TCR diversity within the leptomeninges of AD patients [177]. These data highlight the potential for tissue- and subtype-specific differences in the immunological response of CD8^+^ T cells to antigens. The CD8^+^ T cells with high clonal expansion observed in Gate et al., 2020 [157] in the CSF were GZMK^+^, whereas the ZNF683^high^ subtype detected in the analysis of Hobson et al., 2026 [177] characterized the leptomeninges of AD patients. This tissue specificity was supported by the complete absence of the top leptomeningeal clone in the brains of more than half of the patients with AD analyzed in Hobson et al., 2026 [177], as well as by the identification of tissue-specific clonotypes in the brains of 5×FAD mice, but not in non-transgenic controls [79]. This finding indicates that, while CD8^+^ T cells exhibit a similar pattern of antigen targeting, expanded clones become anatomically segregated between the brain and the leptomeninges in individuals diagnosed with AD. The presence of clonally expanded CD8^+^ T cells associated with AD pathology were confirmed through the implementation of scTCR sequencing on APP23 brain tissue [83]. This analysis unveiled a cluster comprising numerous highly expanded clones (>200 cells), with the maximum frequency of these clones being observed in animals at the late stage of the disease (24 months). Differential gene expression analysis of clonotypes containing more than 200 clones was characterized by T cell exhaustion markers (*Pdcd1*, *Lag3*, and *To*x), which is in line with the findings in 5×FAD mice [101, 162] and indicates that hyperactivation of CD8^+^ T cells might lead to their exhaustion [83].

The potential presence of an antigen that drives the activation and the expansion of CD8^+^ T cells during the progression of AD has recently been observed in APPswe/PSEN1dE9 mice with the expression of GFP used as a measure of antigen-dependent activation. Indeed, the brains of AD mice exhibited elevated numbers of antigen-specific CD8^+^ T cells compared to control mice [178]. To investigate the antigen-specific response and pathogenicity of CD8^+^ T cells in Aβ pathology, APP^NL-G-F^/CD3e^–/–^ mice were adoptively transferred with GFP^+^CD8^+^ cells that have been retrovirally transduced with a TCR sequence identified as being expressed in clonally expanded brain-resident CD8^+^ T cells of APP^NL-G-F^ mice. Interestingly, the adoptively transferred cells reached the brain and accumulated near amyloid plaques, increasing Aβ plaque accumulation compared to control CD8^+^ T cells. These data suggest that the presence of a disease-specific brain antigen is recognized by the TCRs of the CD8^+^ T cells that infiltrate the brains of AD mice [80]. Supporting an antigen-mediated clonal expansion in AD, a recent study that coupled scRNA-seq and flow cytometry analysis revealed a significant increase of MHC-I in the choroid plexus epithelial cells of 5×FAD mice compared with their WT littermates [162]. It has been suggested that Aβ is the most plausible antigen that leads to the infiltration of CD8^+^ T cells into the brain of AD subjects. In fact, immunization of APPswe/PSEN1(L166P) mice via footpad injections with Aβ_33–41_, led to the parenchymal accumulation of CD8^+^ T cells [78]. Similarly, immunization with the Aβ-CoreS RNA vaccine (which expresses human Aβ_1–11_ fused to the hepatitis B surface antigen) increased the number of CD8^+^ T cells in the brains of 5×FAD mice [101], indicating that Aβ is one of the main drivers of CD8^+^ T cell infiltration in the brains of individuals affected by AD. Furthermore, memory APP_(471–479)_-specific KLRG1^+^CD8^+^ T cells accumulated more in the AD brain [179], suggesting a CD8^+^ T cells response to Aβ sequence, as observed in Rosset et al., 2015 [78] and Wang et al., 2024 [101]. Recently, the integration of model predictions with scRNA- and TCR-seq suggested that the most clonally expanded TCRs present in the AD-associated cluster of brain CD8^+^ T cells possess the capacity to interact with MHC complexes that present Aβ peptides [180].

In addition to the mechanisms responsible for the infiltration of CD8^+^ T cells into the AD brain, the contribution of these cells to disease progression remains debated (Table 7). The first attempt to analyze the role of CD8^+^ T cells in AD pathology involved antibody-mediated depletion. The i.p. administration of anti-CD8 antibodies had no impact on spatial learning or behavioral performance in the MWM task, nor on the size or quantity of Aβ plaques in APPswe/PSEN1dE9 mice. However, the treatment increased the expression of genes related to synapses, suggesting that CD8^+^ T cells may influence neuronal function in the brain [156]. Similarly, when 12-month-old APPswe/PSEN1dE9 received an i.v. injection of anti-CD8 antibody, no differences were observed in the total expression levels of Aβ_40_ and Aβ_42_ but only a slight increase in Aβ plaque pathology in the hippocampus. The authors observed a significant difference in Iba1^+^ microglia cells and a higher ratio of reactive (CD68^+^) microglia in the cortex, which is consistent with the data of Unger et al., 2020 [156]. Experiments were performed in parallel with an i.v. injection of WT-derived expanded CD8^+^ T cells. Except for a difference in the Aβ_42_/Aβ_40_ ratio, the pathological outcomes were comparable to those of the animal group that received anti-CD8 antibodies [181]. These results strongly indicate that modulation of CD8^+^ T cells in the periphery does not significantly alter AD-associated pathology in APPswe/PSEN1dE9 mice.

**Table 7:** Studies focusing on targeting CD8^+^ T cells

Animal model	Intervention	Age of the intervention	Effects on the adaptive immune system	End of the experiment	Effects on AD-related pathology	References
APPswe/PSEN1dE9	i.p. injection of anti-CD8 (ten in a month)	12 months	↓ CD8^+^ T cells in peripheral blood and in the brain	13 months	No major effects: Unaltered spatial learning in the MWM test Unaltered Aβ plaques size and number ↑ Iba1^+^ microglia cells ↑ *Trem2* transcript ↑ expression of synapses-related genes	[156]
APPswe/PSEN1dE9	i.v. injection of anti-CD8 (with three doses per week for one month)	12 months	↑ CD8^+^CD69^+^/CD8^+^ T cells ratio, ↑ CD8^+^CD25^+^/CD8^+^ T cells ratio, ↑ CD4^+^CD69^+^/CD4^+^ T cells ratio in the peripheral blood	13 months	Detrimental: ↑ Aβ_42_/Aβ_40_ ratio and ↑ Aβ plaque size in the cortex, and ↑ plaque area and the plaque numbers in the hippocampus ↑ Iba1^+^ cells and ratio of CD68^+^ microglia in the cortex	[181]
APPswe/PSEN1dE9	i.v. injection of WT-derived CD8^+^ T cells	12 months	↓ CD8^+^/CD4^+^ T cells, ↑ CD4^+^CD69^+^/CD4^+^ T cells	13 months	Detrimental: ↑ Aβ plaque area and size in cortex and the hippocampus, but no differences in Aβ_40_ and Aβ_42_ levels ↑ ratio of CD68^+^ microglia in the cortex, but no differences in Iba1^+^ cells	[181]
5×FAD	i.p. injection of anti-CD8 (twice a week for six weeks)	6 months	*NR*	7.5 months	Beneficial: ↑ spatial learning and memory performance in the Barnes maze test ↓ anxiety-related behaviors in the OF and the EPM tests (partially) no changes in the Aβ plaque load ↓ Serpina3n^+^ oligodendrocytes, BCAS1^+^ swellings and EdU^+^Olig2^+^ cells ↓ myelin abnormalities in the cortex ↓ MHC-II^+^Iba1^+^ cells	[40]
5×FAD	i.p. injection of anti-CD8 (six times)	8–10 months	marginal ↓ CD103^+^ and CD11c^+^ CD8^+^cells in the brain ↓ in PD1^+^, IFN-γ^+^, IL10^+^, Lamp1^+^ CD8 T cells	10–12 months	Beneficial: ↓ anxiety response in the OF test and ↑ working memory in the spontaneous alternation test ↓ hippocampal Aβ plaques ↓ Iba1^+^ microglia cells ↓ Nf-H in subiculum, No changes in NeuN^+^ neurons	[101]
APP^NL-G-F^	i.p. injection of anti-CD8 (twice weekly for 2 months)	1 month	↓ CD8^+^ T cells in the brain and the cervical lymph nodes	3 months	Beneficial: ↓ Aβ plaque deposition	[80]
3×Tg-AD	i.p. injection of anti-CD8 (every other day for one month)	6 months	↓ brain CD103^–^CD8^+^ without altering CD103^+^ CD8^+^ T_RM_ cells	7 months	Beneficial: ↑ learning ability in the MWM test and ↑ associative memory in the CFC task ↓ soluble and insoluble pTau in the hippocampus ↓ Aβ load and ↓ levels of insoluble oligomeric and fibrillar Aβ	[163]
APP/PS1 (mutations not specified)	i.v. administration of CD8^+^ T_CM_ (isolated from spleen of WT mice) three times at one week interval	7 months	↑ peripheral CD8^+^ T cells	8.5 months	Detrimental: ↓ spatial learning and memory in the MWM test (but no changes in the OF and the EPM tests) ↑ Aβ deposition	[109]
5×FAD	intranasal CTX administration at two doses (3.75 and 7.5 µg/kg) every other day	4 months	↓ CD45^+^CD3^+^ cells in the brain ↓ CD8^+^/CD4^+^ ratio in the brain ↓ CD8^+^ cell in the hippocampus	6.5 months	Beneficial: ↑ number of target entries and time spent in the target quadrant (only high dose) in the MWM test No changes in Aβ plaque deposition ↓ CXCL9, CXCL10, CXCL16, CCL5, IFN-γ, TNF-α, IL-1β, and IL-6 levels in the brain ↓ Iba1^+^ microglial density and restored ramified morphology with elongated branches and smaller cell bodies ↓ Lamp1^+^ area ↑ total dendritic length and enhanced branching complexity ↑ spine density Restored LTP	[81]
5×FAD	crossbreeding with CXCR6^-/-^	NA	↓ CD8^+^ T cells in the meninges and the brain (due to cell death)	4 and 10 months	Detrimental: ↓ cognitive and memory function in the Y-maze and the NOR tests ↑ Aβ deposits (only in female) ↑ soluble and insoluble Aβ_40_ and Aβ_42_ in the cortex and ↑ soluble Aβ_40_ in the hippocampus ↑ Iba1^+^ microglia and plaque-associated microglia ↑ microglial TNF-α and pro-IL-1β expression	[79]
5×FAD	crossbreeding with B2m^-/-^	NA	altered CD8^+^ cells maturation	4 months	Detrimental: ↓ cognitive and memory function in the Y-maze and the NOR tests ↑ Aβ plaque burden ↑ Iba1^+^ microglia and plaque-associated microglia ↑ microglial TNF-α and pro-IL-1β expression	[79]
P301S	crossbreeding with CD8^KO^	NA	↓ CD8^+^ T cells	7.5 months	Detrimental: ↓ cognitive and motor function in the Y-maze test ↑ pTau spreading ↑ blood levels of tau, GFAP and Nf-L No changes in microglia cells, but ↑ Cd11c^+^MHCII^+^ microglia in the spinal cord	[96]
APP^NL-G-F^	crossdreeding with CD8a^cre^/Rosa26^DTA^	NA	↓ CD8^+^ cell populations in the brain and the cervical lymph nodes	3 months	Beneficial: ↓ Aβ plaque deposition	[80]

The table includes animal models, interventions and the age at which these are implemented (where applicable, otherwise stated as not applicable, NA), the effects on the adaptive immune system, the age at which behavioral tests and sacrifice are performed, and the effects on AD-related pathology. *NR*: not reported

The potentially harmful role of CD8^+^ T cells was further suggested by antibody-mediated depletion in 6-month-old 5×FAD mice. The treated animals exhibited a lower relative escape latency in the Barnes maze test and displayed reverted anxiety-related behaviors. Neuropathological analysis of the brain revealed reduced myelin abnormalities in the cortex, a smaller proportion of disease-associated microglia as well as fewer MHC-II^+^Iba1^+^ cells, without alteration in the amyloid plaque load [40]. The detrimental effects of CD8^+^ T cells were then demonstrated in older 5×FAD mice (8–10-month-old). Animals treated with an anti-CD8 antibody exhibited reduced anxiety in the OF test and improved working memory in the spontaneous alternation test. Anti-CD8 treatment reduced the number of Iba1^+^ microglia cells in the brains of the treated animals, as well as Aβ plaque load and neurofilament heavy chain isoform (Nf-H) [101]. The harmful role of CD8^+^ T cells has recently been confirmed in another animal model of brain amyloidosis. Indeed, i.p. injections of a CD8α-neutralizing antibody reduced Aβ plaque deposition in APP^NL-F-G^ mice [80]. A recent study has revealed that anti-CD8 treatment, which specifically reduces CD103^-^CD8^+^ T_RM_ cells from the brains of 6-month-old 3×Tg-AD mice, enhances cognitive performance in the MWM and the CFC tests. This effect was accompanied by a reduction in Aβ load and pTau levels in the hippocampus of the treated mice [163].

While not the primary focus of the study, a recent article has indicated the detrimental role of CD8^+^ T cells in 5×FAD animals. Indeed, intranasal administration of cobrotoxin (CTX), a short-chain neurotoxin with potent anti-inflammatory and immunomodulatory properties, improved spatial learning and memory in a dose-dependent manner without altering amyloid pathology. The treatment mechanistically suppressed inflammatory chemokines and pro-inflammatory cytokines, reduced microglial activation and dystrophic neurites, and restored synaptic morphology and functional plasticity. CTX also reduced the number of total T cells and CD8^+^ T cells in the brain of the treated animal. This further supports the idea of suppressing CD8^+^ T cells that have infiltrated the brain as a potential target for AD pathology [81].

A few other adoptive transfer experiments provided support for the evidence that CD8^+^ T cells could negatively affect AD pathology. I.v. injection of splenic CD8^+^ T cells into nude mice resulted in their accumulation in the brain and acquisition of a resident memory phenotype. However, these cells unexpectedly caused deficits in learning and working memory, as well as the deposition of Aβ and neurofibrillary tangle (NFT)-like inclusions. Taken together, these findings suggest that CD8^+^ T cells have an intrinsic ability to promote AD-like pathology [179]. This is consistent with the recent finding that transferring CD8^+^ central memory T cells (T_CM_), which are present in high concentrations in the blood of MCI patients, into APP/PS1 mice promoted spatial learning and memory impairments, as well as increasing the amyloid burden [109]. These data suggest that more consistent conclusions about the role of CD8^+^ T cells in AD pathology can only be reached by conducting additional experiments that consider all the different subpopulations of these cells detected in AD patients. Although data from antibody-mediated depletion studies (Kedia et al., 2024 [40], Wang et al., 2024 [101], Terrabuio et al., 2025 [163] and Ohyagi et al., 2025 [80]), adoptive transfer (Panwar et al., 2024 [179], Sun et al., 2025 [109]) and other depleting treatment, as in Li et al., 2026 [81], suggested a detrimental role of CD8^+^ T cells in AD pathology, results from Feng et al., 2023 [181] in which both depletion and adoptive transfer led to similar outcomes, raised concerns about oversimplified conclusions regarding the harmful activities of CD8^+^ T cells in AD progression.

Indeed, experiments performed on CXCR6-depleted mice, which are essential for the tissue-residency phenotype of CD8^+^ T cells [161, 182], support the protective role of these cells during AD progression. 5×FAD/CXCR6^− /−^ mice, which exhibited a reduction in CD8^+^ T cells in the meninges and brain due to increased cell death, showed elevated levels of Aβ deposits, alongside increased concentrations of soluble and insoluble Aβ_40_ and Aβ_42_. Behaviorally, the animals exhibited cognitive impairment in both the spontaneous Y-maze alternation test and the NOR test. Neuroinflammatory processes were also dysregulated in 5×FAD/CXCR6^− /−^ mice, as evidenced by increased TNF-α and IL-1β expression, as well as an increased presence of ameboid-like microglia cells. The authors also reported that 5×FAD mice crossed with animals deficient for β2-microglobulin (5×FAD/B2m^− /−^), which is essential for presenting antigens to CD8^+^ T cells, had diminished abilities to distinguish between arms in the Y-maze test and to discriminate between familiar objects in the NOR test, compared to control 5×FAD mice. Neuropathological analysis revealed increased Aβ deposition and an increase in Iba1^+^ cells [79]. Therefore, a functional antigen presentation process to CD8^+^ T cells, together with the expression of their brain-resident marker CXCR6, is essential in combatting AD pathology. The generation of CD8^+^ T cell-deficient P301S mice indicated a similar neuroprotective function of CD8^+^ T cells in the progression of AD. These animals exhibited increased pTau pathology spread, a higher number of activated microglia, and faster arm-entry decline in the Y-maze test [96].

However, a reduction in Aβ pathology has been observed in APP^NL-F-G^ mice genetically modified to eliminate Cd8a-expressing cells following treatment with diphtheria toxin fragment A [80]. While Su et al., 2023 [79] did not conduct a study focused on the depletion of CD8^+^ cells, Mason et al., 2025 [96] and Ohyagi et al., 2025 [80] crossbred AD mice with transgenic animals that had been specifically depleted of the co-receptor CD8. The contrasting data may again be due to the different AD transgenic mouse models used (P301S vs APP^NL-F-G^, respectively).

### Double-negative T cells

During T cells maturation, some immature thymocytes do not express the classic CD4 nor CD8 co-receptor and become double-negative T (DNT) cells. Although DNT cells represent a small subpopulation in peripheral blood, they play a crucial role in several aspects of both innate and adaptive immune responses [183].

Despite the paucity of research focusing on DNT cells expressing the classic α and β TCR chains in AD, recent research articles have begun investigating them using transgenic mouse models. By crossing APPswe/PSEN1dE9 mice with mice expressing GFP under the Nuclear Receptor Subfamily 4 Group A Member 1 promoter, researchers were able to distinguish between antigen-dependent and antigen-independent activation of DNTs. They found that AD mice exhibited elevated numbers of activated, highly differentiated, antigen-specific DNT cells compared to control animals. This suggests that DNT cells exhibit antigen-dependent immune activity during AD progression [178].

The role of DNT cells in AD has only been investigated using adoptive transfer experiments (Table 8). I.p. injection of DNT cells in APP/PS1 has been shown to impair cognitive function in the MWM test and increase several inflammatory factors, as well as increasing Iba1^+^ and CD11b^+^ cells [184]. Conversely, i.v. administration of DNT cells in 5×FAD mice improved short-term memory and cognitive performance in the NOR and Y-maze tests, as well as spatial learning and memory in the MWM test. The improvement in cognitive ability has been suggested to occur via an increase in dendritic spines and branches, which promote synaptic plasticity. Furthermore, the decrease in the number of plaques in the cortex and hippocampus suggests that DNT treatment has a beneficial effect on 5×FAD mice [185]. The contrasting results of these two studies are due to the use of different animal models and administration routes for the adoptively transferred cells. Nevertheless, further studies are required to investigate the temporal dynamics of DNT cells in the brain parenchyma with the aim of designing more precise experiments and establishing their role in AD pathogenesis.

**Table 8:** Studies focusing on targeting DNT cells

Animal model	Intervention	Age of treatment	Effects on adaptive immune system	End of experiment	Effects on AD-related pathology	References
APP/PS1 (mutations not specified)	I.p. injection of DNT cells (once a week for four times)	7 months	*NR*	8 months	Detrimental: ↓ cognitive function in the MWM test ↑ inflammatory factors (TNF-α, IL-1β and IL-6) ↑ Iba1^+^ and CD11b^+^ cells	[184]
5×FAD	i.v. injection of CD4^+^ T cell-derived DNT cells	6 months	↓ brain expression of *H2-Aa*	7 months	Beneficial: ↑ short-term memory and cognition in the NOR and the Y-maze tests, and ↑ spatial orientation learning and memory ability in the MWM test ↓ small plaques in the cortex and the hippocampus ↓ microglial CD68^+^ signal and transcriptomic changes in microglia (rejuvenated profile) ↓ APOE protein levels ↑ Olig2^+^ cells ↑ IL-1β levels ↑ dendritic spines and more branches at the distal end of their neurites	[185]

The table includes animal models, interventions and the age at which these are implemented (where applicable), the effects on the adaptive immune system, the age at which behavioral tests and sacrifice are performed, and the effects on AD-related pathology. *NR*: not reported

### γδ T cells

γδ T cells adopt an alternative TCR architecture which is different from the predominant subset of CD3^+^ T cells expressing α and β TCR chains and enables these cells to recognize antigens independently of MHC. During development, they can bypass the classic double-positive (CD4^+^CD8^+^) and single-positive (CD4^+^ or CD8^+^) maturation stages and exit the thymus as DNT lymphocytes [186].

Although the function of γδ T cells in AD has not been thoroughly investigated so far, some clonotypes expressing TCRγ are more prevalent in the brains of AD patients than in those of control subjects [187]. Similarly, spectral flow cytometry analysis on single-cell suspension of hippocampal tissue from old APPswe/PSEN1dE9 mice (22–28 months of age) revealed an increased number of γδ T cells in comparison to WT mice [165].

Furthermore, an increased number of γδ T cells, which are a major source of IL-17, were found in the meninges of female 3×Tg-AD mice. Interestingly, i.c.v. delivery of anti-IL-17 reduced the locomotion impairments and anxiety in the OF and in the elevated plus maze (EPM) tests, respectively. This treatment also prevented short-term memory impairment in the Y-maze test and decreased long-term potentiation without affecting Aβ plaques and pTau levels [188]. As previously observed in literature [189, 190], these data suggested the beneficial effect of IL-17 depletion on cognition, independent of AD pathology. These outcomes are comparable when the role of IL-17 is investigated using a genetic model. Crossbreeding APPswe/PSEN1(L166P) mice with IL-17a^−/−^ (APP^tg^/IL17a^−/−^) resulted in a fewer Aβ deposits in both the cortex and hippocampus, compared to control mice. APP^tg^/IL17a^−/−^ mice had fewer and shorter microglia branches with fewer intersections [191].

These studies suggested that γδ T cells, particularly those that produce IL-17, appear to exacerbate AD pathology. However, further research is required before these cells can be considered a viable therapeutic target for AD.

### Mucosal-associated invariant T cells

Mucosal-associated invariant T (MAIT) cells are a type of innate-like T cell that can recognize bacterial and fungal metabolites derived from vitamin B biosynthesis via their TCRs when they are presented by MHC class I-related molecule (MR1) [192, 193]. Despite their prevalence in mucosal sites, the lungs and the peripheral blood, MAIT cells have also been found in the mouse brain [194]. Their contribution to AD and other CNS disorders has only recently been recognized [195, 196]. Indeed, MR1 protein expression has been observed to increase in the brains of individuals diagnosed with AD, particularly within Iba1^+^ microglia cells [197]. Furthermore, the present study found this protein to be expressed in the CA1 region of the hippocampus in 8-month-old 5×FAD mice. This was accompanied by an increase in the total number of MAIT cells (from 6 months in the cortex and from 8 months in the hippocampus) and activated MAIT cells (CD69^+^ and CD25^+^) (Table 9). Moreover, in 5×FAD mice lacking MR1 and consequently also lacking MAIT cells, as their development requires MR1-expressing thymocytes [200], the incidence of Aβ plaque pathology is reduced relative to that observed in regular 5×FAD mice [197]. Further experimentation on 8-month-old 5×FAD knockout mice for MR1 revealed a reduction in markers of dystrophic neurites, a decrease in insoluble Aβ_40_, and an increase in PSD95 levels in the hippocampus [198]. Collectively these data suggest a detrimental role of the MR1/MAIT cell axis in the development of AD pathology.

**Table 9:** Studies focusing on targeting MAIT cells

Animal model	Intervention	Age of treatment	Effects on adaptive immune system	End of experiment	Effects on AD-related pathology	References
5×FAD	Crossbreeding with MR1^-/-^	NA	*NR*	2 to 8 months	Beneficial: ↓ Aβ plaques at 2 and 6 months of age in the hippocampus, at 6 and 8 months of age in the temporal cortex No changes in Iba1^+^ microglia number ↓ Lamp1 and ubiquitin (at 8 months of age in the hippocampus), and n-terminal APP (also at 6 months of age in the hippocampus and cortex) levels ↓ insoluble Aβ_40_, but not changes in Aβ_42_ levels ↓ *Lamp1, App* and *Psap*, and ↑ *Mapt* from scRNA-seq analysis of hippocampus ↓ hippocampal NeuN at 6 months of age ↑ PSD95 in the hippocampus (at 8 months of age) No changes in SYP	[197, 198]
P301S	Crossbreeding with MR1^-/-^	NA	Absence of MAIT cells in the meninges	7–8 months	Detrimental: ↑ pTau pathology (at 7 and 8 months of age) ↓ hippocampus weight and volume (at 7.5 months of age) ↑ leakage in the meningeal barrier (at 7 months of age) ↑ Iba1^+^ cells in the hippocampus (at 7 months of age) ↑ levels of TNF, CD68 and F4/80 in microglia (at 7 months of age) ↑ percentages of a microglia subset expressing high levels of proinflammatory genes from snRNA-seq analysis (at 7 months of age) ↑ *Apoe*, *Apoc1*, *Spp1* and *Rsad2* in hippocampal microglia cells (at 7 months of age) ↑ IL-1β, IL-1α, and TNF-α in the brain homogenate (at 7 months of age)	[199]

The table includes animal models, interventions and the age at which these are implemented (where applicable, otherwise stated as not applicable, NA), the effects on the adaptive immune system, the age at which behavioral tests and sacrifice are performed, and the effects on AD-related pathology. *NR*: not reported

Conversely, the role of MAIT appears to be protective in pure tauopathy. A recent study revealed an increased number of MAIT cells in the meninges of 7-month-old P301S mice, but not in brain parenchyma. 7.5-month-old P301S mice lacking MAIT cells exhibited increased pTau pathology and hippocampal atrophy. It is hypothesized that MAIT cell deficiency may exacerbate neurodegeneration in P301S mice via meningeal leakage and increased microglial inflammation. The adoptive transfer of MAIT cells into MR1^−/−^ P301S mice was found to significantly repress pTau pathology and hippocampal atrophy, prevent meningeal leakage, and reduce Iba1 and TNF expression [199].

Further experimentation is necessary to ascertain the role of MAIT in the progression of AD, particularly by performing adoptive transfers of MAIT cells into amyloidogenic animal models. Nevertheless, these cells have the potential to become a valuable therapeutic target for AD, given their capacity to preserve neuronal function and protect the meninges following reactive oxidative damage [201].

## Immunomodulation in AD

In consideration of the pivotal role of immune function in AD, there is an ongoing focus on the development of innovative therapeutic methodologies that target innate immune cells or molecules with an immunological function towards microglia or peripheral myeloid cells (see for review [202]). Nevertheless, in recent decades, there has been a marked increase in the level of interest in the field of AD-associated adaptive immune system abnormalities. As a result, immunomodulatory strategies, which were previously only used in cancer and autoimmune disease therapies, are now being exploited to combat AD [202, 203]. These include the previously mentioned Treg-based strategies [95, 128, 130–140, 142] and innovative combinatorial immunomodulatory approaches (see for review [204]).

### Immunomodulation via checkpoint inhibition

Even though the precise mechanism remains the subject of ongoing debate, the prevailing view at present is that innate and adaptive immune system exhaustion is associated with the pathology of AD [205–207]. Consequently, researchers in this field have proposed boosting immune activity systemically or alleviating adaptive immune system suppression as potential therapeutic approaches for AD [32].

Immune checkpoint inhibition primarily involves preventing the interaction between programmed cell death protein 1 (PD-1) and its ligand (PD-L1). PD-1 is expressed on the surface of T cells, and when it interacts with PD-L1, it leads to the suppression of T cell survival, proliferation, and activity by interfering with the TCR signaling cascade [208]. The aim of this therapeutic strategy is to boost T cell-mediated immune responses and enhance the body’s ability to combat threats [209].

Administering a single dose of anti-PD-1 and anti-PD-L1 via i.p. injection to a tauopathy mouse model (expressing two mutations associated with genetic cases of frontotemporal dementia, K257T/P301S) reduced cognitive deficits when the animals were tested in Y- and T-maze tasks (Table 10). One month later, neuropathological analysis revealed decreased tau deposition and aggregation. Furthermore, the treated animals exhibited reduced levels of inflammatory cytokines and increased numbers of surviving neurons. Similarly, a single dose of anti-PD-L1 treatment improved the performance of 5×FAD mice in the RAWM test. Neuropathological analysis revealed reduced amyloid burden and astrogliosis, as well as increased SYP levels [210]. Delayed cognitive decline, as assessed using the RAWM test, was observed in 5×FAD mice that had received anti-PD-L1 treatment on three occasions. Analysis of brain tissue revealed reduced neuronal loss, evidenced by an increase in NeuN^+^ cells and a reduction in Caspase-3 signals [210].

**Table 10:** Studies focusing on immune-checkpoint inhibitor therapies

Animal model	Intervention	Age of the intervention	Effects on the adaptive immune system	End of the experiment	Effects on AD-related pathology	References
K257T/P301S	i.p. injection of anti-PD1 and anti-PD-L1 (single dose)	6–8 months	↑ effector memory T cells (CD44^+^CD62L^low^)	7–9 months	Beneficial: ↑ short-term spatial memory in the T-maze test and ↑ performance in the Y maze test ↓ inflammatory cytokines (*Tnf, Il6, Il12p40,* and *Il1b*) ↓ pTau (Thr212, Ser214 and Thr231) levels and ↓ tau aggregation ↑ surviving neurons ↑ monocytes-derived macrophages (CD45^high^CD11b^high^) recruitment to the brain	[210]
5×FAD	i.p. injection of anti-PD-L1 or anti-PD-1 (once)	6 months	*NR*	7 months	Beneficial: ↑ spatial learning and memory performance in the RAWM test ↓ amyloid burden ↓ astrogliosis ↑ levels of SYP	[210]
5×FAD	i.p. injection of anti-PD1 (three times over two months)	3.5 months	*NR*	6.5 months	Beneficial: ↑ spatial learning and memory performance in the RAWM test ↑ neuronal survival (↑ NeuN and ↓ Caspase-3 signal)	[210]
APPswe/PSEN1dE9	i.p. injection of anti-PD1 (two at three-days interval)	8 or 15 months	*NR*	9 or 16 months	Beneficial: ↓ Aβ plaques	[211]
5×FAD	i.p. injection of anti-PD1 (two at three-days interval)	10 months	*NR*	11 months	Beneficial: ↑ cognitive performance in the RAWM test ↑ brain CD45^+^CD11b^+^ cells ↑ *Ifng* and *Ccl2* transcript levels ↓ astrogliosis ↓ Aβ plaques	[211]
P301S/human-APOE4-knock-in	i.p. injection of anti-PDCD1 (every five days)	8 months	↑ Foxp3^+^CD4^+^ and PDCD1^+^Foxp3^+^CD4^+^ Treg cells in the brain	9.5 months	Beneficial: ameliorated brain atrophy ↓ pTau levels	[86]
K257T/P301S	i.p. injection of anti-PD-L1 (once every 6 weeks, over four months)	6–7 months	*NR*	10–11 months	Beneficial: ↑ cognitive performance in the T-maze test	[212]
JNPL3	I.p. injection of anti-PD-1 (every week for twelve weeks)	10–11 months	*NR*	14–15 months	No major effects: ↑ locomotor activity in the OF test (but no effect in the rotarod test) No changes in cognitive tests (object recognition, spontaneous alternation, novel location, Barnes maze and fear conditioning tests) No consistent changes in soluble and sarkosyl insoluble tau fractions	[213]
5×FAD	i.p. injection of anti-PD-1 and anti-CTLA-4 (twice a week for six weeks)	6 months	↑ CD8^+^ T cells in the cortex	7.5 months	Detrimental: ↑ anxiety-related behaviors in the OF and the EPM tests No changes in the amyloid plaque load ↑ number of Serpina3n^+^ oligodendrocytes and EdU^+^Olig2^+^ cells ↑ myelin abnormalities in the cortex ↑ MHC-II^+^Iba1^+^ cells	[40]
P301S	i.p. injection of anti-PD-1 and anti-TIGIT (every other day for up to eight weeks)	4 months	Shifted distribution of spinal cord CD8^+^ T cells into the gray matter	5 months	Detrimental: ↓ cognitive and motor functions in the Y-maze test ↑ ratio of multinucleated Iba1^+^ myeloid cells in the gray versus white matter	[96]
APP/PS1(M146L)	i.p. injection of a chimeric anti-PD1 antibody (with murine fragment crystallizable region, two injections at three days interval, for two times)	8 months	↑ IFN-γ expressing CD4^+^ and CD8^+^ T cells in the splenocytes ↑ total number of CD11b^+^ myeloid cells in the brain	10 months	No major effects: Unaltered Aβ plaque burden in the hippocampal and cortical regions No changes in Aβ_40_ and Aβ_42_ levels in the brain	[214]
APP/PS1(A246E)	i.p. injection of anti-PD1 (same antibody as Baruch et al., 2016 [211], two injections at three days interval, for two times)	11 months	*NR*	13 months	No major effects: Unaltered Aβ plaque burden in the hippocampal and cortical regions No changes in Aβ_40_ and Aβ_42_ levels in the brain	[214]
PDAPP	i.p. injection of a chimeric anti-PD1 antibody (with murine fragment crystallizable region, two injections at three days interval, for three times)	18 months	*NR*	21 months	No major effects: Unaltered Aβ plaque burden in the hippocampal and cortical regions No changes in Aβ_42_ levels in the brain	[214]

The table includes animal models, interventions and the age at which these are implemented (where applicable), the effects on the adaptive immune system, the age at which behavioral tests and sacrifice are performed, and the effects on AD-related pathology. *NR*: not reported

However, chronic neurodegenerative conditions such as AD may require prolonged immune checkpoint blockade. The beneficial effects of immune checkpoint inhibition were observed following two i.p. injections of an anti-PD1 antibody at three-day intervals. Researchers demonstrated a clear reduction in Aβ plaques in 8- and 15-month-old APPswe/PSEN1dE9 mice, which were sacrificed one month after treatment commenced. Similarly, continuous treatment with an anti-PD-1 antibody improved the cognitive performance of 5×FAD mice in the RAWM test and reduced Aβ plaques [211]. Similar beneficial effects were obtained with anti-PDCD1 (PD-1) i.p. injections, every five days for one-and-a-half month, in P301S/human-APOE4-knock-in mice. This treatment decreased tau-mediated neurodegeneration and the pTau signal in brain tissue [86].

Similar cognitive improvements were observed in K257T/P301S mice undergoing chronic immune checkpoint inhibition. This involved the mice receiving i.p. injections of an anti-PD-L1 antibody at six-weekly intervals for four months. As early as one month after treatment began, the mice showed a stronger preference for the novel arm of the T-maze test [212]. Treatment with anti-PD-1/PD-L1 antibodies has been shown to mechanistically increase the infiltration of monocyte-derived macrophages into the brain (CD45^high^CD11b^high^) [210, 211, 215, 216] and of immunosuppressive CD4^+^Foxp3^+^ Tregs [86, 217]. This suggests that the main beneficial effect of this treatment is to activate the peripheral immune system, allowing immune repair cells (such as Tregs) to enter the brain [32]. However, one study revealed a modest increase in locomotor activity in JNPL3 tauopathy mice (carrying the P301L mutation, a variant of MAPT gene associated with frontotemporal dementia) treated with an anti-PD-1 antibody weekly for twelve weeks with no significant impact on cognition or tau pathology [213]. This suggests that differences in the animal model and treatment regimen used in Baruch et al., 2020 [212] and Rosenzweig et al., 2019 [210], can influence the outcome of therapy. Furthermore, the combination of an anti-PD-1 antibody and an anti-cytotoxic T lymphocyte-associated protein 4 (CTLA-4) antibody, intended to enhance immune checkpoint blockade therapy, exacerbated anxiety-related behaviors in 5×FAD mice without altering the amyloid plaque load in their cortex. Furthermore, the combined treatment increased the number of MHC-II^+^Iba1^+^ cells and infiltrating CD8^+^ T cells, and exacerbated myelin abnormalities [40]. Similarly, detrimental effects of checkpoint inhibition were observed in P301S mice treated with a combination of anti-PD-1 and anti-T-cell immunoreceptor with immunoglobulin and immunoreceptor tyrosine-based inhibitory motif domains (TIGIT) antibodies, resulting in an accelerated decline in motor functions [96]. Although Baruch et al., 2016 [211], Baruch et al., 2020 [212], Rosenzweig et al., 2019 [210] and Chen et al., 2023 [86] found that long-term treatment with immune-checkpoint inhibitors was beneficial, Kedia et al., 2024 [40] and Mason et al., 2025 [96] suggested that a controlled intervention is necessary to ameliorate AD-like pathology in transgenic animal models.

However, concerns have been raised regarding the potential consequences of immune tolerance disruption through PD-1 checkpoint inhibition. Indeed, a study utilizing three distinct animal models of AD (APP/PS1(M146L) and APP/PS1(A246E), which produce amyloid deposits of significant, compact nature, and PDAPP, expressing the APP gene with the V717F mutation and characterized by more diffuse deposits) failed to support the hypothesis that inhibition of PD-1 would result in a reduction in amyloid pathology [214]. Therefore, despite the maintenance of the treatment regimen, discrepancies between animal models may nevertheless influence the reproducibility of the beneficial effect of PD-1 inhibition as a treatment for AD. Furthermore, loss of PD-1 expression has been shown to increase Aβ plaque deposition and diminish microglial Aβ uptake in APPswe/PSEN1dE9 mice [218]. These findings emphasize that the same immune mechanism can be both protective and detrimental in AD, indicating the need for cell-targeted approaches to regulating neuroinflammation rather than generalized ones.

### Immunomodulation via glatiramer acetate

Glatiramer acetate (GA) is a mixture of random polymers composed of glutamic acid, alanine, tyrosine and lysine, which have been shown to polarize T cells towards a Th2 phenotype [219]. Although GA has been approved for the treatment of MS, it has been reported that there are cutaneous and hepatic side effects [220, 221]. GA has also been posited as a potential therapeutic intervention for the management of AD.

In APPswe/PSEN1dE9 animals, subcutaneous (s.c.) GA injection improved spatial learning and memory in the MWM test, increased neurogenesis, and decreased Aβ plaque formation and the number of CD11b^+^ microglia [222] (Table 11). In a similar manner, weekly injections of GA in the same animal model at 10 months of age resulted in a slight reduction in memory and learning dysfunction in the Barnes maze test. Brain neuropathology revealed strong decreased cerebral Aβ levels and astrocytosis, further confirming the beneficial effect of GA treatment [223]. These favorable outcomes were also recapitulated in 3×Tg-AD mice. After two months of s.c. GA injections, treated mice performed better in the NOR test. Neuropathological analysis of the animals revealed reduced Aβ deposits, decreased soluble and insoluble Aβ_42_, and fewer NFTs [224]. To enhance the beneficial effects of Tregs on AD pathology, GA was injected subcutaneously into APPswe/PS1dE9 mice. The treatment improved spatial learning and memory abilities in the MWM and the Y-maze tests and reduced Aβ plaque burden, as well as the number of Iba1^+^ microglia cells. However, no mitigating effects on Aβ-related pathology were observed in older animals (6-month-old) with established amyloid deposition when treatment resulted in a decrease in Tregs in the spleen, as opposed to the effect found in younger animals [225]. This further supports the importance of choosing the right therapeutic window for immunomodulatory treatment in AD. However, it has been demonstrated that, while reducing the proportion of splenic Tregs, weekly injections of GA improved cognitive performance and reduced deposition of Aβ plaques in 5×FAD mice [142]. These contradictory results regarding the Tregs-mediated mechanism of GA’s beneficial effects on AD pathology, as reported by Huang et al., 2024 [225], and Baruch et al., 2015 [142], suggest that, while the fine-tuning of the adaptive immune system with immunomodulators such as GA is a promising therapeutic approach for treating AD, further research is needed to understand its mechanism of action. A more recent analysis was conducted on 5×FAD mice at various stages of the disease and revealed a slight enhancement in Aβ accumulation in female 5×FAD mice at 8.5 months of age [226]. This finding is in contrast with the data presented by Baruch et al., 2015 [142] (where, however, a shorter therapeutic regimen was used), which emphasizes the necessity for future experiments to incorporate sex as a pivotal variable in evaluating the impact of GA on the progression of AD pathologies.

**Table 11:** Studies focusing on GA treatment

Animal model	Intervention	Age of the intervention	Effects on the adaptive immune system	End of the experiment	Effects on AD-related pathology	References
APPswe/PSEN1dE9	s.c. injection of GA (twice per week and then once a week for one month)	8 months	Stimulated autoreactive T cells ↑ CD3^+^ cells associated with Aβ plaques	9.5 months	Beneficial: ↑ spatial learning and memory abilities in the MWM test ↑ neurogenesis (BrdU^+^ and DCX^+^ neurons) ↓ Aβ plaques formation ↓ CD11b^+^ microglia	[222]
APPswe/PSEN1dE9	s.c. injection of GA (once a week over a period of two months)	10 months	*NR* NOTE: ↑ cerebral recruitment of CD45^hi^Iba1^+^ macrophage and CD11b^+^ CD45^high^/Ly6C^high^ monocytes	12–13 months	Beneficial: ↓ latency and incorrect box entries in the Barnes maze test ↓ 4G8^+^ and Thio-S ^+^ Aβ plaques ↓ insoluble Aβ_40_ and Aβ_42_ ↑ hippocampal presynaptic VGluT1 ↓ GFAP^+^ cells No changes in brain MCP1 levels ↑ IL-10 levels	[223]
3×Tg-AD	s.c. injection of GA (weekly for two months)	15 months	Unaltered splenocytic CD4^+^ T cell proportions	17 months	Beneficial: ↑ cognitive performance in the NOR test ↓ Aβ deposits and both soluble and insoluble Aβ_42_ ↓ pTau (at S396 and S404), but ↑ pTau T205 Unaltered microglia complexity (but upregulated *Gm1070* and *Dcstamp* in hippocampal CD11b^+^CD45^int^ cells)	[224]
APPswe/PSEN1dE9	s.c. injection of GA (twice per week and then once a week for three months)	2 months	↑ splenic Tregs (CD4^+^CD25^+^Foxp3^+^) expansion NOTE: ↓ in 6-month-old mice treated for three months	5 months	Beneficial: ↑ spatial learning and memory abilities in the MWM and the Y-maze tests ↓ Aβ plaques and both Aβ_40_ and Aβ_42_ ↓ Iba1^+^ microglia ↑ *Il4* and *Tgfb1* transcripts in the brain NOTE: no changes in 6-month-old mice treated for three months	[225]
5×FAD	s.c. injection of GA (weekly, five injections over one month)	7 months	↑ leukocyte trafficking via choroid plexus ↓ splenocyte Foxp3^+^ Treg levels	8 months	Beneficial: ↑ cognitive performance in the RAWM test ↓ Aβ plaques NOTE: daily regimen of GA did not show any effects	[142]
5×FAD	s.c. injection of GA (twice during the first week of treatment and then once a week for seven additional weeks)	2.5, 5.5 and 8.5 months	*NR*	4.5, 6.5 and 10.5 months	No major effects: ↑ 6E10^+^ Aβ plaques (in mice treated at 8.5 months of age) ↑ Iba1^+^ microglial colocalization with Aβ plaques (in mice treated at 5.5 months of age) No effect on MeX04^+^ core and LAMP1^+^ dystrophic neurites No changes in microglial levels of P2Ry12, TMEM119, CD11c and Clec7a	[226]

The table includes animal models, interventions and the age at which these are implemented (where applicable), the effects on the adaptive immune system, the age at which behavioral tests and sacrifice are performed, and the effects on AD-related pathology. *NR*: not reported

## Lesson learned from active immunization

The knowledge acquired regarding the role of the innate immune system in AD has established the foundation for contemporary therapeutic approaches that leverage the activity of the adaptive immune system. Passive immunization is a process that provides immediate but temporary protection. In AD, it involves the injection of pre-made antibodies against Aβ [111, 227, 228] or tau [229] (see for review [230]). Active immunization, on the other hand, stimulates the adaptive immune system to produce antibodies using AD-related immunogens, including different Aβ and tau-derived peptides (see for review [231, 232]). Notwithstanding the fact that passive immunization represents the most advanced treatment for AD, there is a growing interest in active immunization.

Pioneering research has shown that intramuscular vaccination with human Aβ_42_ can prevent plaque deposition in 2-month-old mice and reduce amyloid plaques in 13–15- and 18-month-old PDAPP mice [233]. Subsequently, a behavioral test involving 7.5-month-old APP/PS1(M146L) mice demonstrated that subcutaneous injection with human Aβ_42_ maintains intact working memory [234, 235]. Similar promising behavioral and neuropathological data were obtained through the immunization of the Aβ_42_ peptide in its β-pleated sheet conformation in the TgCRND8 mouse model of AD (which expresses a mutant human APP transgene containing the K670N, M671L, and V717F mutations) [236]. Remarkably, both studies found that the mice vaccinated with the Aβ vaccine developed antibodies against Aβ. Since the early 2000s, active immunization using vaccines based on the full-length Aβ_42_ peptide has been shown to have beneficial effects in various AD animal models, both alone [237–246], and in combination with adjuvants designed to enhance the immunogenicity of the vaccines [247–253]. Notably, positive effects have also been observed with oral immunization using food expressing Aβ_42_ peptide [254, 255].

A one-year phase 1 clinical trial of AN-1792, an experimental Aβ_42_-based vaccine developed by Elan (Dublin, Ireland) and Wyeth (Madison, NJ, USA), revealed no signs of toxicity or adverse events in human subjects. However, the follow-up phase 2 trial was terminated early when four patients developed symptoms consistent with meningoencephalitis [256–258]. Further neuropathological analysis revealed that some subjects who received the vaccination experienced brain volume shrinkage [259], while others developed cerebral amyloid angiopathy [260]. Although these reactions were not identified in preclinical safety and efficacy testing, one study suggests that autoimmune encephalomyelitis can be induced in wild-type mice that are vaccinated with the Aβ peptide [261]. Nevertheless, subsequent analysis of a non-human primate model suggested that Aβ immunotherapy could effectively treat AD. Indeed, ten months of immunization with the Aβ peptide reduced cerebral plaque deposits [262]. Furthermore, AN-1792 vaccine antibody responders exhibited cognitive improvements, as well as reduced levels of CSF tau [263] and amyloid plaques in the frontal cortex, without evidence of meningoencephalitis or leukoencephalopathy [264]. Interestingly, analysis of two case reports from the AN-1792 vaccine trial indicated that meningoencephalitis may have been triggered by an autoimmune response involving T cells. Post-mortem examinations of their brains revealed infiltration of T lymphocytes in the leptomeninges, cerebrovasculature, and cerebral neocortex [265].

Analysis of peripheral lymphocytes exposed to peptide-derived Aβ sequences revealed that, in humans, CD4^+^ T cells recognize more C-terminal epitopes [65], and that, in mice, they are responsive to the central region of the Aβ peptide (residues 7–30), depending on their haplotypes [266, 267]. Notably, a single immunization with Aβ 10–24 resulted in T cell-mediated encephalitis without eliciting the production of Aβ antibodies [266]. Conversely, the Aβ 33–41 peptide region has been suggested as a potential epitope recognized by CD8^+^ T cells [78]. Fine epitope analysis of anti-Aβ antibodies generated in Aβ-immunized mice identified region 1–15 as a dominant B-cell epitope [268]. Similarly, anti-Aβ antibodies from immunized monkeys [262] and humans [269], were found to recognize an epitope located within the amino terminus of the Aβ protein. Therefore, to reduce the detrimental infiltration of T cells into the brains of immunized individuals mediated by Aβ, several studies have suggested active immunization with N-terminal peptides. Positive outcomes were indeed observed with the use of the 1–6 [270–272], 3–6, [273], 3–10 [274–284], 1–10 [285], 4–10 [286], 1–11 [287–293], 1–14 [294–296], 1–15 [297–316] Aβ peptide regions. Nevertheless, an active vaccine targeting the C-terminal end has also been tested. Phase I clinical trials of ABvac40 (consisting of multiple repeats of a synthetic peptide corresponding to amino acids 33–40 of Aβ_40_) revealed no amyloid-related imaging abnormalities indicating vasogenic edema or sulcal effusions, and no microhemorrhages were detected in immunized patients [317]. A subsequent Phase 2 clinical trial revealed that ABvac40 induced a specific and sustained immune response in plasma, with detectable antibodies present in the CSF. The number of cases of amyloid-related imaging abnormalities (ARIA) was comparable between the vaccine and placebo groups. No ARIA-edema or meningoencephalomyelitis events were detected [318]. These data suggest that the mechanism guiding T cell infiltration and the subsequent detrimental effects on the brain parenchyma of patients with AD require further analysis to understand the exact epitope responsible for such immune system response.

Several attempts have been made to exploit tau-mediated active immunization [319–338] and some strategies are currently being considered for clinical trials, including AADvac1 [339–341], ACI-35.030 [342] and AV-1980R (NCT07158905). There have yet to be any reports of an aberrant T cell-mediated response to tau epitopes. It can be hypothesized that since most of these strategies are designed using short, targeted tau sequences consisting of B cell epitopes (such as the 30-amino-acid sequence centered around the pathological HXPGGG motif of AADvac1 or the N-terminal Tau 2–18 peptide used in the AV-1980R vaccine) they selectively elicit anti-tau antibodies and avoid the activation of tau-specific autoreactive T cells. Comparable positive effects on cognition have been observed following active immunization with a combination of Aβ and tau epitopes [343].

While the immunological mechanism responsible for T cell infiltration following immunization with AD-associated peptides remains to be analyzed, these vaccination strategies have demonstrated the pivotal role of the adaptive immune system in the development of novel AD therapies.

## Conclusion

Further experimentation is required to elucidate the immunological mechanisms and functions that result in the infiltration of adaptive immune system cells into the brain of the AD subjects. Fortunately, there are emerging innovative approaches to identify the antigens responsible for CD8^+^ and CD4^+^ T cell parenchymal recruitment. These approaches include profiling the brain’s MHC peptidome (the collection of MHC-binding peptides), using tetramer screening techniques to identify antigen-specific T cells within brain samples, and predicting antigens using fine-tuned prediction models based on TCR sequences [344].

As the activation status and proportion of different adaptive immune cells change during the progression of the disease, and aging has been identified as one of the most significant risk factors for AD, longitudinal studies utilizing crossbreeding less artificial animal models of AD (e.g., late-onset AD models from the MODEL-AD consortium) with conditional transgenic models to delete and activate a specific gene involved in the maturation of a particular adaptive immune cell subpopulation in adulthood (e.g., CRE reporter mice), or adoptive transfer using specific disease-associated cell populations (using Thy1.2^+^ and Thy1.1^+^ mice to easily distinguish the donor’s transferred cells from the host’s native cells), or their depletion with antibodies or inhibitors precisely targeting the population of interest will facilitate a more profound comprehension of the neuroprotective or pathologically prone nature of specific adaptive immune cell subpopulations. Furthermore, it will be crucial to perform loss- and gain-of-function analysis in the same experimental setting to increase the robustness of specific findings related to the adaptive immune system cell type studied.

**Fig. 1:**
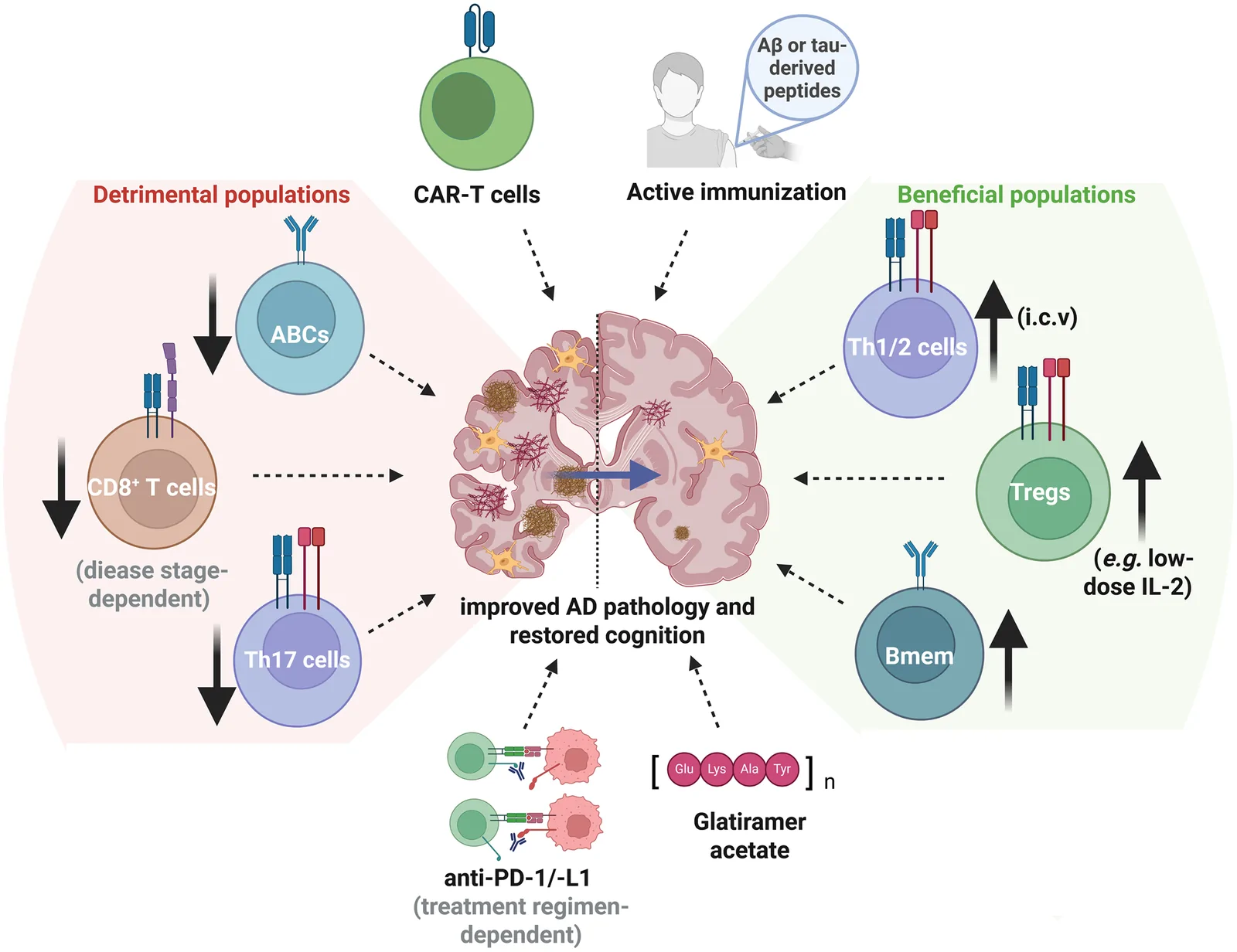
Therapeutic interventions targeting the adaptive immune system for Alzheimer’s disease. Whilst Th17 cells, ABCs and CD8^+^ T cells (albeit in a time-specific manner, as developmental deletion appears to be detrimental) appear to exacerbate the progression of AD, Bmem, Tregs and Th1/2 may have beneficial effects on AD pathology, including promoting plaque clearance and neurofibrillary tangle reduction, and improving cognitive function. General B, CD3^+^ and CD4^+^ T cells populations, Tfh, DNT, γδ T and MAIT cells (not represented in the infographic) require further analysis to define their role in AD. Promising approaches to ameliorating AD pathology include novel CAR-T cell-based therapies, active immunization with Aβ- or tau-derived peptides, boosting the immune system with checkpoint inhibitors (e.g., by adjusting the anti-PD-1/PD-L1 therapy regimen), and modulating the responses of specific adaptive immune cell subsets using glatiramer acetate or other cell-targeting strategies (e.g., expanding the Treg population with low-dose IL-2; infusing Th1/2 cells intracerebroventricularly; or reducing CD8^+^ T cells once the disease has become established). Abbreviations: age-associated B cells (ABCs); alanine (Ala); chimeric antigen receptor (CAR); CD4^+^ regulatory T cells (Tregs); CD4^+^ helper T cells (Th); glutamic acid (Glu); intracerebroventricular (i.c.v.); lysine (Lys); memory B (Bmem); tyrosine (Tyr). *created with BioRender.com*

In conclusion, while the role of certain adaptive immune cells, including B lymphocytes, CD4^+^ T helper, DNT, γδ T and MAIT cells, requires further exploration before they can be considered as therapeutic targets for AD pathology, the role of other adaptive immune cells (i.e., CD4^+^ Tregs) in AD is well defined and they are already being considered in several AD clinical trials. However, novel therapeutic applications that aim to target specific subsets of T cells (including Th cells and CD8^+^ T cells), B lymphocytes (by expanding Bmem cells and reducing ABCs), or the adaptive immune response (by using checkpoint inhibitors, GA and active immunization) must be precisely tailored to the progression of AD in order to achieve beneficial therapeutic outcomes (Fig. 1).

## Data Availability

No datasets were generated or analysed during the current study.

## Abbreviations

ABCs: Age-associated B cells
AD: Alzheimer’s disease
ARIA: Amyloid-related imaging abnormalities
ATRA: All-trans retinoic acid
AAV: Adeno associated virus
Aβ: Amyloid β
BAFF: B-cell activating factor
BBB: Blood–brain barrier
Bmem: memory B cells
CAR: Chimeric antigen receptor
CFC: Contextual fear conditioning
CNS: Central nervous system
COX-2: Cyclooxygenase 2
CSF: Cerebrospinal fluid
CTLA-4: Cytotoxic T lymphocyte-associated protein 4
CTX: Cobrotoxin
CXCL: Chemokine (C-X-C motif) ligand
CXCR: Chemokine (C-X-C motif) receptor
DCX: Doublecortin
DNT: Double-negative T
ELS: Ectopic lymphoid structures
EPM: Elevated plus maze
GA: Glatiramer acetate
GFP: Green fluorescent protein
GZM: Granzyme
Ig: Immunoglobulin
IL: Interleukin
ISGs: interferon-stimulated genes
i.c.v.: Intracerebroventricular
i.p.: Intraperitoneal
i.v.: Intravenous
Lamp1: lysosomal-associated membrane protein 1
LTP: long-term potentiation
MCI: Mild cognitive impairment
MHC: Major Histocompatibility Complex
MR1: MHC class I-related molecule
MS: Multiple sclerosis
MWM: Morris Water Maze
NFT: Neurofibrillary tangle
Nf-H: Neurofilament heavy chain
Nf-L: Neurofilament light chain
NOR: Novel object recognition
OF: Open field
PCs: Plasma cells
PD-1: Programmed cell death protein 1
PD-L1: PD-1 ligand
PSD95: Postsynaptic density protein 95
pTau: Phosphorylated tau
RAWM: Radial arm water maze
scRNA-seq: single-cell RNA sequencing
snRNA-seq: single-nucleus RNA sequencing
SYN: Synapsin
SYP: Synaptophysin
s.c.: Subcutaneous
TCR: T cell receptor
TEMRA: effector memory T cells that re-express CD45RA
T_CM_: Central memory T cells
Tfh: Follicular Th
Th: Helper T cells
Thio-S: Thioflavin S
TIGIT: T-cell immunoreceptor with immunoglobulin and immunoreceptor tyrosine-based inhibitory motif domains
TNF-α: Tumor necrosis factor α
TNFSF10: Tumor necrosis factor ligand superfamily member 10
Tregs: Regulatory T cells
VCAM-1: Vascular cell adhesion molecule 1
WT: Wild type
